# Three month inhalation exposure to low-level PM_2.5_ induced brain toxicity in an Alzheimer’s disease mouse model

**DOI:** 10.1371/journal.pone.0254587

**Published:** 2021-08-26

**Authors:** Sheng-Han Lee, Yi-Hsuan Chen, Chu-Chun Chien, Yuan-Horng Yan, Hsin-Chang Chen, Hsiao-Chi Chuang, Hui-I Hsieh, Kuan-Hung Cho, Li-Wei Kuo, Charles C. -K. Chou, Ming-Jang Chiu, Boon Lead Tee, Ta-Fu Chen, Tsun-Jen Cheng

**Affiliations:** 1 Institute of Environmental and Occupational Health Sciences, College of Public Health, National Taiwan University, Taipei, Taiwan; 2 Department of Pathology, Kaohsiung Municipal Ta-Tung Hospital, Kaohsiung Medical University Hospital, Kaohsiung Medical University, Kaohsiung, Taiwan; 3 Department of Endocrinology and Metabolism, Kuang Tien General Hospital, Taichung, Taiwan; 4 Department of Nutrition and Institute of Biomedical Nutrition, Hung Kuang University, Taichung, Taiwan; 5 Institute of Food Safety and Health, College of Public Health, National Taiwan University, Taipei, Taiwan; 6 Department of Public Health, National Taiwan University, Taipei, Taiwan; 7 School of Respiratory Therapy, College of Medicine, Taipei Medical University, Taipei, Taiwan; 8 Department of Occupational Medicine, Cathay General Hospital, Taipei, Taiwan; 9 Institute of Biomedical Engineering and Nanomedicine, National Health Research Institutes, Miaoli, Taiwan; 10 Institute of Medical Device and Imaging, National Taiwan University College of Medicine, Taipei, Taiwan; 11 Research Center for Environmental Changes, Academia Sinica, Taipei, Taiwan; 12 Department of Neurology, National Taiwan University Hospital, Taipei, Taiwan; 13 Department of Neurology, Memory and Aging Center, University of California at San Francisco, San Francisco, California, United States of America; Torrey Pines Institute for Molecular Studies, UNITED STATES

## Abstract

Although numerous epidemiological studies revealed an association between ambient fine particulate matter (PM_2.5_) exposure and Alzheimer’s disease (AD), the PM_2.5_-induced neuron toxicity and associated mechanisms were not fully elucidated. The present study assessed brain toxicity in 6-month-old female triple-transgenic AD (3xTg-AD) mice following subchronic exposure to PM_2.5_ via an inhalation system. The treated mice were whole-bodily and continuously exposed to real-world PM_2.5_ for 3 months, while the control mice inhaled filtered air. Changes in cognitive and motor functions were evaluated using the Morris Water Maze and rotarod tests. Magnetic resonance imaging analysis was used to record gross brain volume alterations, and tissue staining with hematoxylin and eosin, Nissl, and immunohistochemistry methods were used to monitor pathological changes in microstructures after PM_2.5_ exposure. The levels of AD-related hallmarks and the oxidative stress biomarker malondialdehyde (MDA) were assessed using Western blot analysis and liquid chromatography-mass spectrometry, respectively. Our results showed that subchronic exposure to environmental levels of PM_2.5_ induced obvious neuronal loss in the cortex of exposed mice, but without significant impairment of cognitive and motor function. Increased levels of phosphorylated-tau and MDA were also observed in olfactory bulb or hippocampus after PM_2.5_ exposure, but no amyloid pathology was detected, as reported in previous studies. These results revealed that a relatively lower level of PM_2.5_ subchronic exposure from the environmental atmosphere still induced certain neurodegenerative changes in the brains of AD mice, especially in the olfactory bulb, entorhinal cortex and hippocampus, which is consistent with the nasal entry and spreading route for PM exposure. Systemic factors may also contribute to the neuronal toxicity. The effects of PM_2.5_ after a more prolonged exposure period are needed to establish a more comprehensive picture of the PM_2.5_-mediated development of AD.

## Introduction

Particulate matter (PM) has become a major health risk for humans because of its widespread presence in our surroundings. A smaller particle in the PM family, fine particulate matter (PM_2.5_), induces more severe adverse effects than larger particles. In addition to the particle size-dependent toxicity, the chemical composition, such as metal, polycyclic aromatic hydrocarbons, sulfate, and nitrate, may also play an important role in PM-induced toxicity [1]. Numerous studies, including our previous studies, showed PM-induced pulmonary and cardiovascular adverse effects [2–7]. Recent epidemiological studies revealed that PM exposure was associated with decreased brain volume, cognitive decline, abnormal blood brain barrier, brain neuronal inflammation, and an increased risk of Alzheimer’s disease (AD), Parkinson’s disease, and ischemic cerebrovascular disease [8–10].

AD is a common cause of dementia, and chronic PM exposure may contribute to the occurrence, development, and/or exacerbation of this neurodegenerative disorders. Current epidemiological studies demonstrated that PM exposure positively correlated with the increased risk of AD [11–16]. However, the detailed mechanisms of PM-induced neurotoxicity on AD are not clear.

The senile plaques and neurofibrillary tangles are typical pathological hallmarks of AD and derive from the accumulation of the pathological amyloid β-peptides (Aβ) and breakdown of the hyperphosphorylated microtubule-associated protein tau. Previous studies showed that subchronic or chronic exposure to PM_2.5_ or diesel exhaust nanoparticles increased Aβ expression and deposits in mouse or rat brains [17–19]. In addition to Aβ and tau pathology, perturbation of inflammation, oxidative stress, and microglia activation were also observed in the PM- and air pollutant-exposed rodent brains or neuronal cell culture [17, 20, 21]. Although previous studies demonstrated that PM played an important role in AD development and progress, some scientific gaps in the complete picture of PM-induced AD toxicity remain. First, numerous animal studies focused on toxic effects after concentrated or higher-dosed ambient particle exposure, and limited studies examined the real effects underlying the environmental level of PM_2.5_ exposure. Compared to more studies on PM-induced neuronal toxicity in wild-type rodent strains, the PM_2.5_-mediated toxicity effects in AD transgene mouse strains are not well known. Therefore, the present study examined the neuronal toxicity and its related mechanisms after subchronic, real-world exposure to PM_2.5_ using a customized inhalation system.

## Materials and methods

### Animals

The current study was carried out in strict accordance with the recommendations in the Guide for the Care and Use of Laboratory Animals of the National Institutes of Health. The protocol was approved by the Institutional Animal Care and Use Committee of the College of Medicine and the College of Public Health, National Taiwan University (Permit Number: 20160545). All surgery was performed under isoflurane and every effort was made to minimize the stress, pains, and suffering of the experimental animals. Three-week-old AD transgenic mice (3xTg-AD mice) were obtained from Prof. Chiu’s team, acclimated and inbred with mice from Jackson Laboratory (Bar Harbor, ME, USA). These mice were housed in individual ventilated cages with a 12-h light/dark cycle and maintained at a constant temperature (22±2°C) and relative humidity (50±5%). Mice had free access to LabDiet® 5001 (PMI® Nutrition International, Brentwood, MO, USA) and water during the study.

### PM_2.5_ exposure system

**Fig 1:**
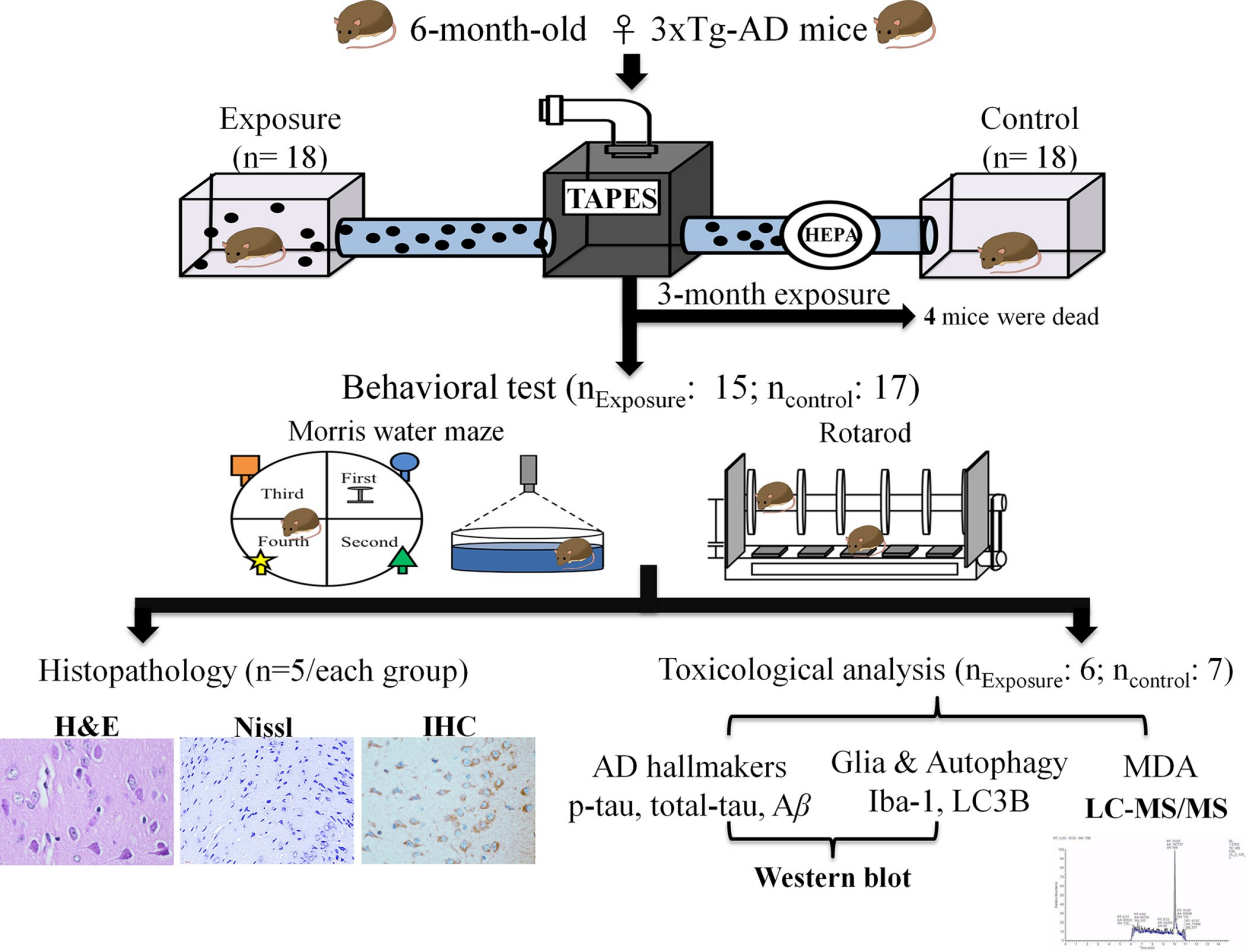
The experimental framework for PM_2.5_ exposure in AD mice.

The customized PM_2.5_ exposure system, also called the Taipei Air Pollutant Exposure System (TAPES), was used to perform the current study (Fig 1). The detailed structure and materials of this whole body, real-world PM_2.5_ exposure system are described in our previous publication [22]. Briefly, this system was located at the College of Public Health, National Taiwan University (Taipei, Taiwan). Outdoor ambient air was continuously sampled and introduced into the whole-body exposure chambers, and the environment inside simulated the exposure condition in the real world. The exposure group inhaled directly introduced ambient air, and high-efficiency particulate air-filtered air was provided for the control group. The mass concentrations and particle sizes were monitored and calculated using 37-mm Teflon filters (Pall Corporation, Port Washington, New York, USA) and the DustTrakTM II Aerosol Monitor 8530 (TSI, Shoreview, Minnesota, USA). Component analyses of PM_2.5_ were performed using ion chromatography [23] and inductively coupled plasma mass spectrometry [24].

### Experimental design

The entire experimental framework is shown in Fig 1. Six-month-old female 3xTg-AD mice were continuously exposed to PM_2.5_-containing air (n = 18) or particle-free air (n = 18) for three months during the Winter of 2018 to the Spring of 2019. After a three-month exposure, the mice were subjected to behavioral tests (n_exposure_ = 15; n_control_ = 17), including the Morris Water Maze (MWM) and rotarod tests. All mice were performed MWM test initially. After accomplishment of MWM test, mice were subsequently subjected to the rotarod test. During the experimental period, the health condition of mice, including weight changes, skin condition, and mobility were monitored at least every two days. At the conclusion of experimental period, mice were deeply anesthetised in 5% isoflurane. The anesthetised mice were perfused with saline or euthanised by rapid decapitation to collect the brain tissue for further histopathological examination (n = 5/each group) and toxicological analysis (N_exposure_ = 6; N_control_ = 7).

### Morris Water Maze test

The MWM test is a standard method to evaluate memory and spatial learning ability in animal models. The procedure of the MWM test was described previously [25], with some modifications. Briefly, the entire system was composed of a maze (100-cm diameter pool) and an animal behavior/trajectory tracking analysis system (Noldus EthoVision 3.1, The Netherlands). The pool was filled with water (maintained at 24±1°C) that reached a depth of 30 cm. This test was performed using an acquisition phase (the first to the fourth days) followed by a probe trial (the fifth day). In the acquisition phase, a platform, which was 1 cm below the water surface, was placed in the center of the northeast quadrant of the maze (Fig 1). In the acquisition phase, each mouse was trained to find the platform from each quadrant of the maze three times. If the mouse failed to find the platform in 60 seconds, then it was placed on the platform for 10 seconds. In the probe trail, mice were placed into the pool and forced to swim without the platform for 60 seconds. The escape latency, distance moved, and swimming velocity during the acquisition phase and the swimming velocity, time spent in the target quadrant, and crossing numbers in the target quadrant of the probe trail were recorded for further analyses.

### Rotarod test

The rotarod test was performed based on a previous study [26]. The Rotarod treadmill system (UGO47600, Ugo Basile Biological research apparatus, Gemonio (VA), Italy) was used to evaluate the athletic ability, balance and motor coordination of mice (Fig 1). Each mouse was trained three times daily for three consecutive days during the adaptation period. The training speed was set at a constant speed (4 rpm) over a period of 6 min to make the mouse familiar with the roller and avoid dropping from the system. In the experimental period, each mouse was placed on a rotating rod with an initial speed (4 rpm) to a maximum speed (40 rpm) using an acceleration of 1 rpm/10 sec for a 6-min period. The time and speed of staying on the rotating rod were recorded when the mouse dropped onto the sensor. Each mouse was assessed three times daily for three consecutive days. The average time of these trials from each mouse was calculated as the final result.

### Sample collection

After behavior tests, some mice underwent a series of histopathological examinations (n = 5/each group). The remaining mice were sacrificed (N_exposure_ = 6; N_control_ = 7) for toxicological analysis, including Western blot analysis for AD-associated protein expression levels and analyses of malondialdehyde (MDA) to evaluate oxidative stress. The collected brains from each mouse were separated into cortex, hippocampus, olfactory bulb, and cerebellum and stored -80°C until further analysis.

### Histopathological and immunohistochemical staining

The collected mouse brains were trimmed, fixed in 10% neutral buffered formalin, dehydrated, and embedded in paraffin wax. The paraffin-embedded tissues were sectioned at a thickness of 3–5 μm and stained with hematoxylin and eosin (H&E) and cresyl violet (Nissl stain). A certified pathologist and a well-trained medical doctor confirmed the histopathological results and number of neurons in a blinded manner. A representative image for neuronal cell counting in an equal brain region is shown in (S1 Fig in S1 File), and the region of interest method was performed to count neural cell density in the hippocampus (S2 Fig in S1 File).

For immunohistochemical (IHC) staining, caspase 3 and ubiquitin were conducted to study the effects of neuronal apoptosis, whereas TNFα and Iba-1 were used for neuroinflammation and microglia activation. All sections were subjected to antigen retrieval in boiling citrate buffer (pH 6.0) for 20 mins. After a blocking step, tissue sections were stained with primary antibodies, including caspase 3 (1:500, bs-0081R, Bioss, USA), ubiquitin (1:100, E3350, Spring Bioscience, USA), TNFα (1:100, bs-2081R, Bioss, USA), and Iba-1 (1:200, BS90680, Bioworld, USA) followed by an anti-rabbit HRP-conjugated secondary antibody (TA00C2, BioTnA, Kaohsiung, Taiwan). The results were recorded using the TAlink mouse/rabbit polymer detection system (TAHC04D, BioTnA, Kaohsiung, Taiwan). All IHC slides were screened and digitized using a Motic Easyscan Digital Slide Scanner and DSAssistant and EasyScanner software (Motic Hong Kong Limited, Hong Kong, China).

### Western blot analysis

The details of Western blot analysis are referenced in previous studies [27, 28] with some modifications. Equal brain tissue (15 mg) from each sample was homogenized in 60 μl of a protein lysis solution. After vortexing and centrifugation (4°C, 12,000 rpm for 20 min), the supernatants of mixed solutions were collected and stored in -80°C until further analysis. The total protein levels from each sample were measured using a BCA Protein Assay Kit. Equal protein solutions from each lysed brain tissue sample were electrophoresed using sodium dodecylsulfate polyacrylamide gel electrophoresis (SDS-PAGE) and transferred onto polyvinylidene fluoride membranes. A deionized water solution with 5% skim milk was used for membrane blocking. After blocking, the processed membranes were individually incubated with different primary antibodies, including beta amyloid 1–42 (Aβ_42_; ab201060; 1:1000), total-tau (t-tau; GTX112981; 1:1000), phosphorylated-tau (p-tau; GTX24864; 1:1000), LC3B as the autophagosome marker (LC3B (D11) XP^@^ Rabbit mAb, #3868; 1:1000), Iba-1 as the marker of neuroinflammation and microglia activation (Iba-1; GTX100042; 1:1000), and beta-actin (GTX629630; 1:10000), purchased from GeneTex (Irvine, CA, USA), Abcam (Cambridge, UK), and Cell Signaling Technology (Danvers, MA, USA), overnight at 4°C. After washing, the membranes were incubated with anti-rabbit (1:10000) or anti-mouse (1:10000) of horseradish peroxidase-labeled secondary antibodies purchased from GeneTex (Irvine, CA, USA). Enhanced chemiluminescence (ECL) was used for the immunoreaction, and the image results were acquired using the BioSpectrum 810 Imaging System (UVP, Upland, CA, USA). The protein expression levels were calculated using Image-Pro version 4 (Media Cybernetics, Inc., MD, USA).

### MDA assessment

The level of MDA in each brain area measured using a liquid chromatography-tandem mass spectrometry (LC-MS/MS) system, as previously described [29]. Initially, equal amounts (10 mg) of brain tissue samples were mixed with 250 μl of potassium chloride aqueous solution (1.15%, w/v) and homogenized. A sodium hydroxide aqueous solution (6 M, 100 μl) was added to the homogenized samples and incubated for 45 min at 60°C. Subsequently, 250 μl acetonitrile solution (100%) was added to the mixed solutions, vortexed and centrifuged (25°C, 15,000 rpm for 10 min). The supernatants of the mixed solutions were collected and reacted with a 2,4-dinitrophenylhydrazine (DNPH) solution (5 mM) to form the MDA-DNPH derivatives for LC-MS/MS analysis.

The LC-MS/MS system was a Thermo Scientific Accela™ chromatographic system coupled with a TSQ Quantum™ Access mass spectrometer (Thermo Fisher Scientific Inc., Waltham, WA, USA). Reversed-phase chromatography was performed on a Thermo Syncronis C18 column (4.6 mm × 150 mm, 5.0 μm) with a binary solvent system, including mobile phase A: acetonitrile containing 0.1% (v/v) acetic acid, and mobile phase B: Milli-Q water containing 0.1% (v/v) acetic acid. The selected reaction monitoring mode with a precursor ion ([M+H]^+^ = *m/z* 235) and two product ions (*m/z* = 116 and 131) were used to detect the MDA-DNPH. The levels of MDA were assessed using Xcalibur 2.2 software (Thermo Fisher Scientific Inc., Waltham, WA, USA).

### Statistical analyses

All statistical analyses were performed using SAS 9.4 software (SAS Institute, Cary, NC, USA). The Wilcoxon rank sum test was used for group comparison. The level of significance was set at *p* < 0.05.

## Results

### The concentration and chemical composition of PM_2.5_

The mean mass concentration of PM_2.5_ during the exposure period was 11.38 μg/m^3^ (with a range from 6.14 to 17.49). The chemical composition of PM_2.5_ is shown in Table 1 and (S1 and S2 Tables in S1 File). The most abundant water-soluble ions were SO_4_^2-^ (65.99%), NH_4_^+^ (21.63%), and NO_3_^-^ (7.89%)_,_ and Na, K, Ca, Zn, Fe, Al, and Mg accounted for 90% of the metal content of PM_2.5_ composition. Notably, the heavy metals are more soluble in water than the crystal elements (shown in S1 and S2 Tables in S1 File).

**Table 1:** The water-soluble ion composition of the PM_2.5_ around exposure period.

	Mean (μg/m^3^)	Median (μg/m^3^)	SD (μg/m^3^)	Min (μg/m^3^)	Max (μg/m^3^)
Na^+^	0.1	0.1	0.0	0.0	0.1
NH_4_^+^	1.5	1.5	0.5	0.7	2.3
K^+^	0.1	0.1	0.0	0.0	0.3
Mg^2+^	0.0	0.0	0.0	0.0	0.0
Ca^2+^	0.0	0.0	0.0	0.0	0.1
Cl^-^	0.0	0.0	0.0	0.0	0.0
NO^3-^	0.6	0.4	0.5	0.2	1.4
SO_4_^2-^	4.7	4.6	1.5	2.0	6.5

### Body weight and mortality

There was no significant difference in body weight between the PM_2.5_-exposed (n = 15) and control groups (n = 17) during the experimental period (S3 Fig in S1 File). However, three mice in the exposure group and one mouse in the control group died. The mortality rate of the exposure group (20%) was higher than the control group (6%) after the 3-month PM_2.5_ exposure (Fig 1).

### Behavior performance

Escape latencies in the MWM test were decreased on the 3rd and 4th days compared to the 1st day in the PM_2.5_-exposed (n = 15) and control mice (n = 17) in the acquisition phase, but there were no significant differences in escape latency, distance moved, or swimming velocity between the two groups (S4 Fig in S1 File). Only the percentage of time spent in the target quadrant in the probe trial was slightly higher in the exposed group, but there were no significant changes in other parameters (S4 Fig in S1 File). The values of latency to fall and speed at fall in the rotarod test were higher in the exposed group compared to the control group on 2nd day, without a significant difference (S5 Fig in S1 File).

### Histopathology

**Fig 2:**
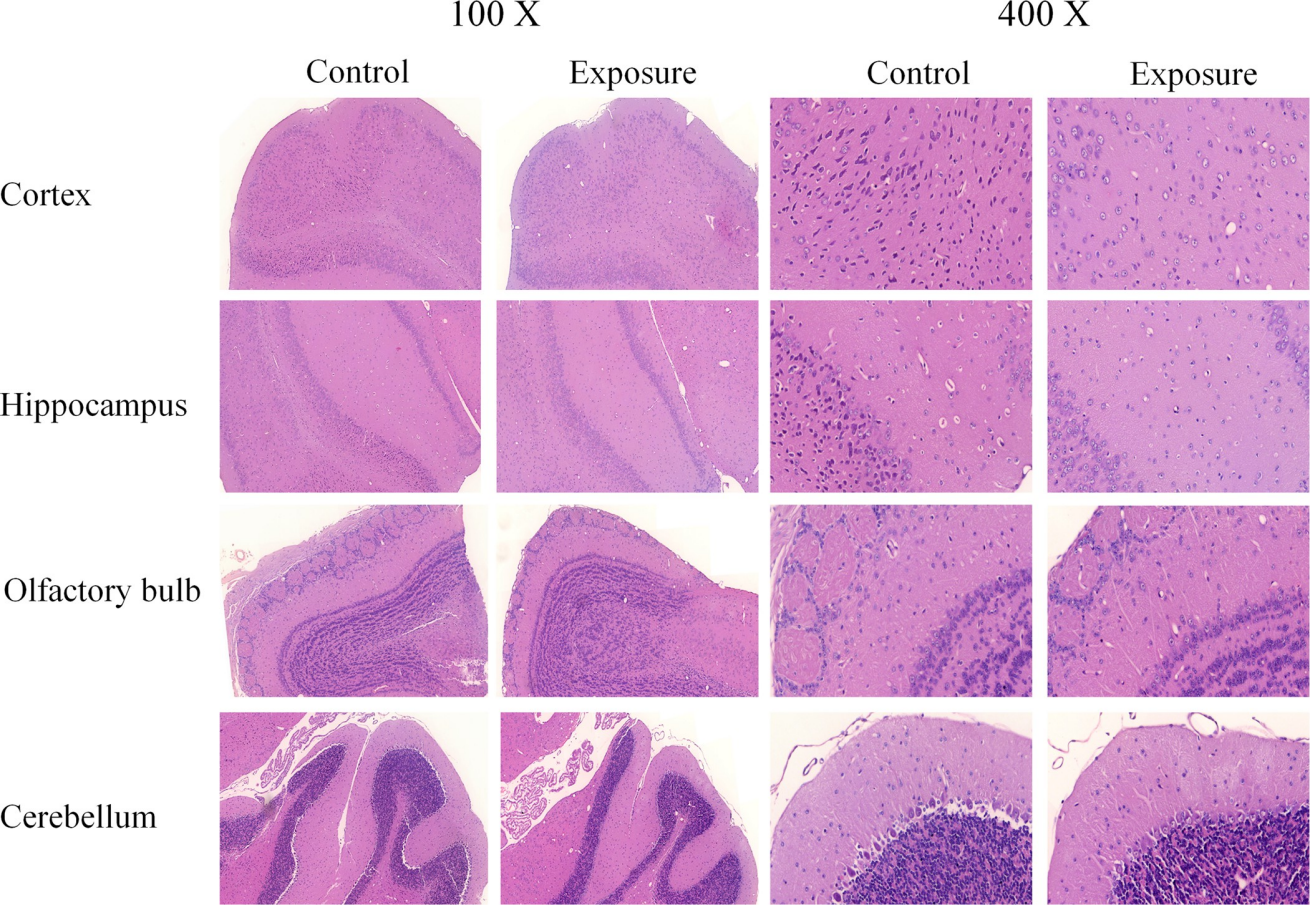
The histopathological views of H&E staining (100X & 400X) in AD mouse brain after PM_2.5_ exposure.

**Fig 3:**
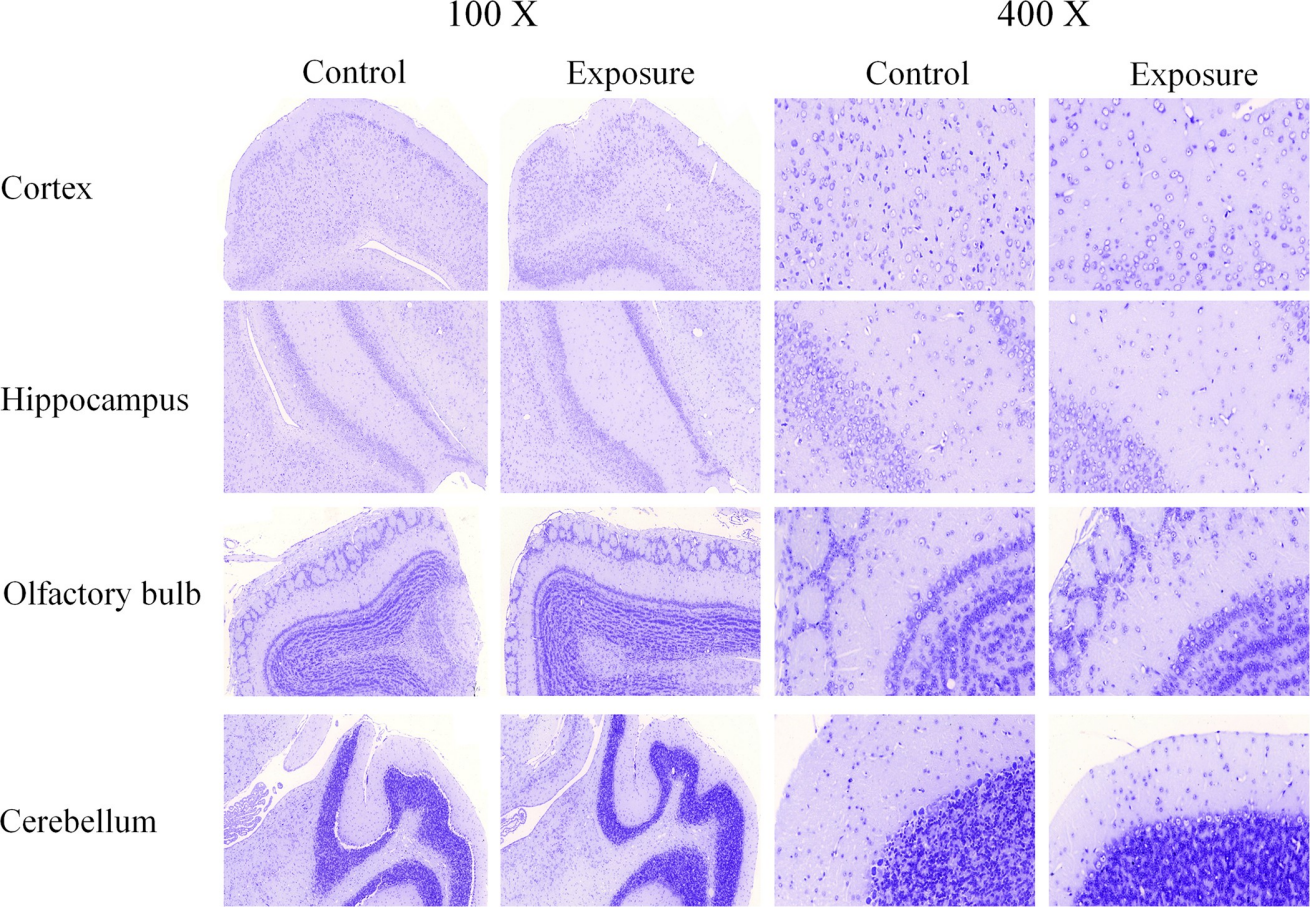
The histopathological views of Nissl staining (100X & 400X) in AD mouse brain after PM_2.5_ exposure.

**Fig 4:**
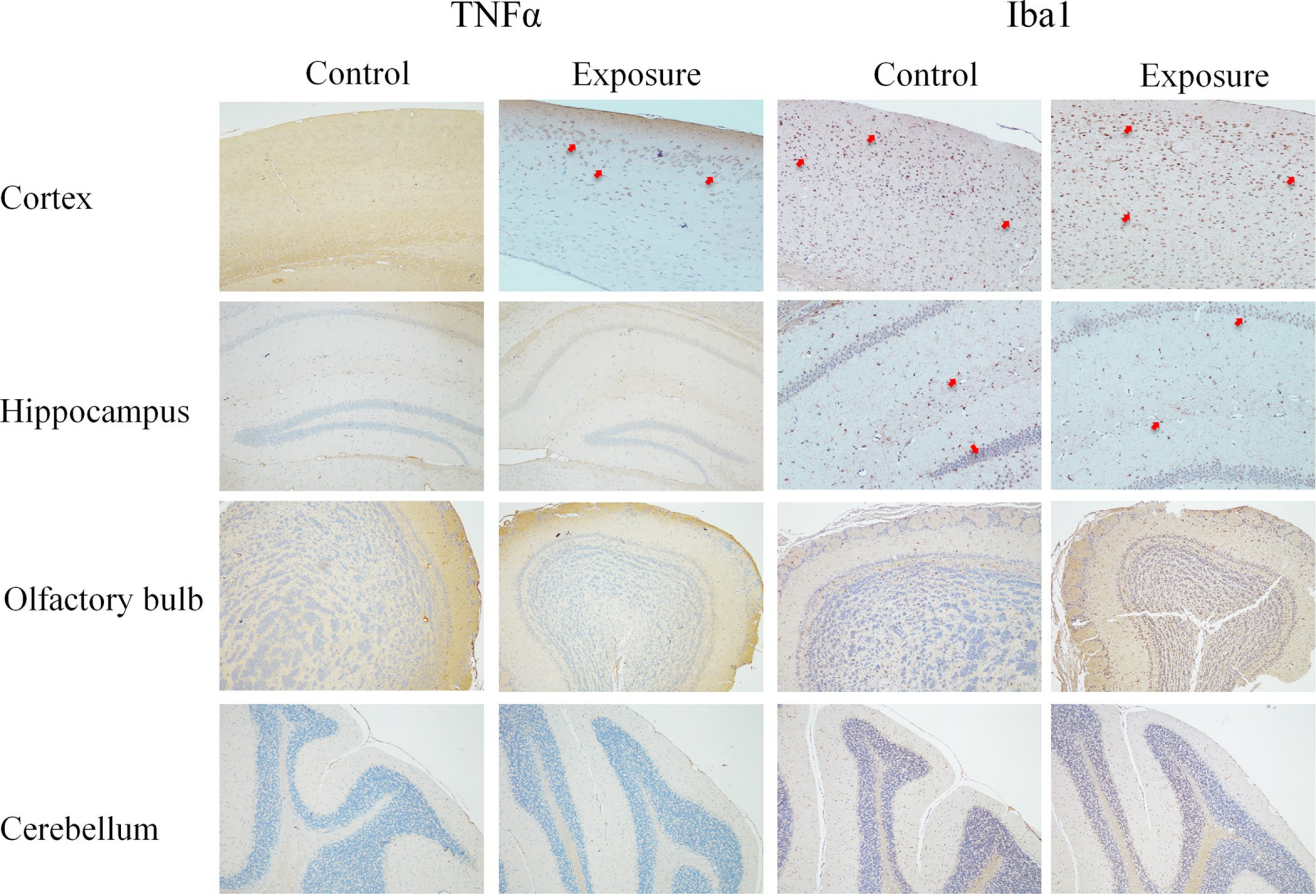
The histopathological views of IHC staining (100X & 400X) of TNFα and Iba-1 in AD mouse brain after PM_2.5_ exposure. The red arrow indicates the representative IHC positively stained neurons.

The histopathological results from the H&E and Nissl stains in each mouse brain area after 3-months of exposure are shown in Figs 2 and 3 and Table 2. The numbers of neurons were significantly decreased in the cortex of AD mice in the exposure group (n = 5) compared to the control group (n = 5) with both stains (Table 2), but no obvious neuron loss was found in the hippocampus, olfactory bulb, or cerebellum. Notably, a similar finding was also detected in the entorhinal cortex. Several neuronal morphological changes were also found in the cortex (especially in the entorhinal cortex) of some PM_2.5_-exposed mice. Granulovacuolar degeneration (GVD) was observed in the H&E (S6a Fig in S1 File) and Nissl staining of one exposed mouse (S6d Fig in S1 File), and lipofuscin (LF) was found in the H&E staining of another exposed mouse (S6b Fig in S1 File). IHC revealed obvious positive staining of TNFα and Iba-1 in certain brain sections of AD mice (Fig 4 and S7 Fig in S1 File), but not caspase 3 or ubiquitin. However, there were no significant differences between the two groups.

**Table 2:** The numbers of neuronal cells in the cortex sections after 3-month exposure of PM_2.5_.

Brain section	Control^a^	Exposure^a^	*p*-value
H&E			
Whole Cortex	1410 ± 841	213 ± 178	0.016
Entorhinal cortex	622 ± 381	155 ± 156	0.032
Nissl staining			
Whole Cortex	1755 ± 592	628 ± 236	0.016
Entorhinal cortex	856 ± 311	323 ± 111	0.016

^a^ values are representative as mean ± SD of neuronal numbers

### Western blot analysis

**Fig 5:**
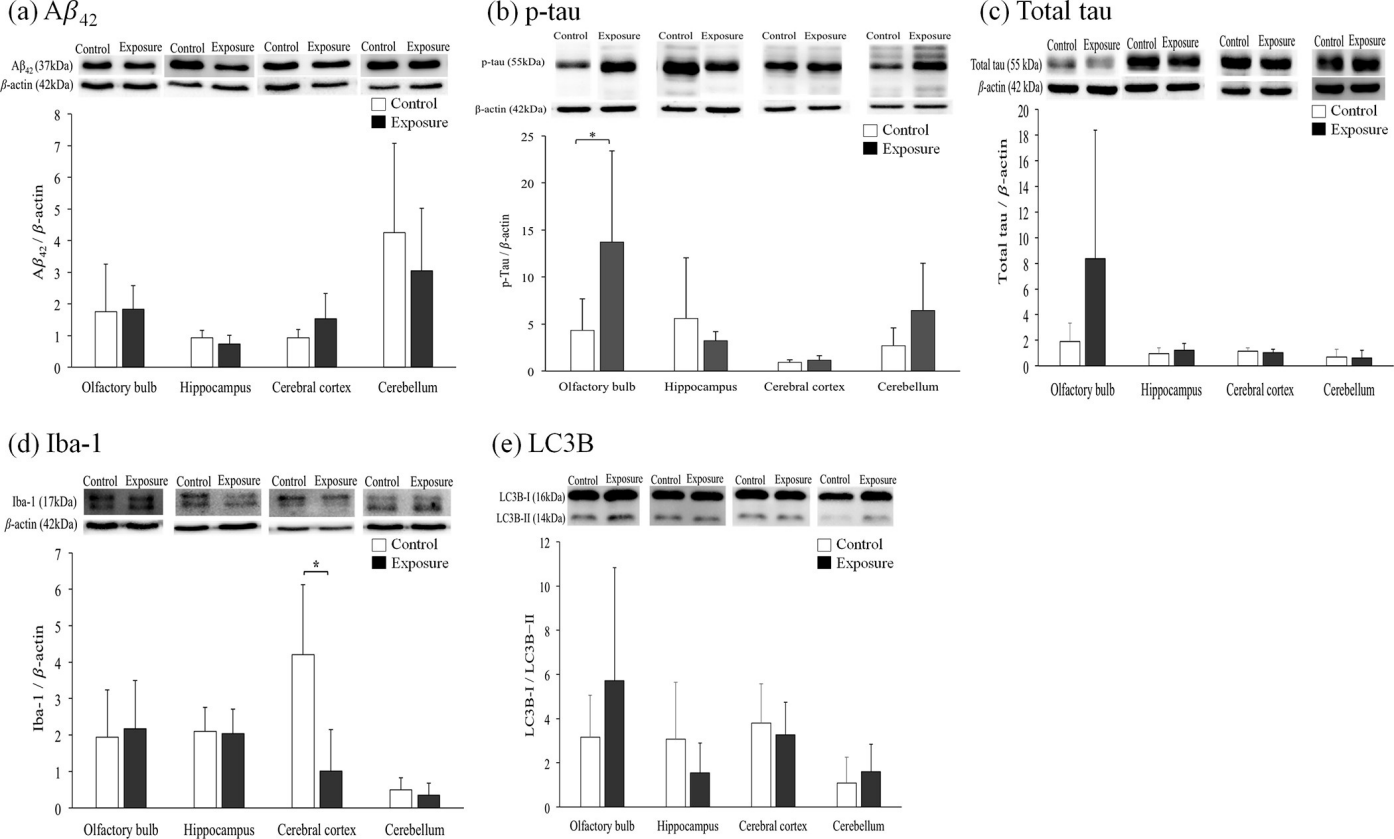
The levels of AD-related protein expression (a) p-tau, (b) total tau, (c) Iba-1, (d) Aβ_42_, and (e) LC3B in AD mouse brains after PM_2.5_ exposure (n = 6) compared to control mice (n = 7). The values are presented as mean *±* SD. * indicates the significant differences between two groups with a *p* value lower than 0.05.

To evaluate AD-related neuronal toxicity, different expression levels of certain AD-related hallmarks, such as Aβ_42_, t-tau, and p-tau, and the autophagosome marker (LC3B) and neuroinflammation marker (Iba-1) in the cortex, hippocampus, olfactory bulb, and cerebellum of mice in the PM_2.5_-exposed (n = 6) and control groups (n = 7) after 3 months of exposure are shown in Fig 5. Increased p-tau protein levels in the olfactory bulb and decreased Iba-1 protein in the cortex were found in the exposure group compared to the control group. No significant changes in the Aβ_42_, t-tau, or LC3B proteins were observed in the cortex, hippocampus, olfactory bulb, and cerebellum of exposed mice.

### MDA level

**Fig 6:**
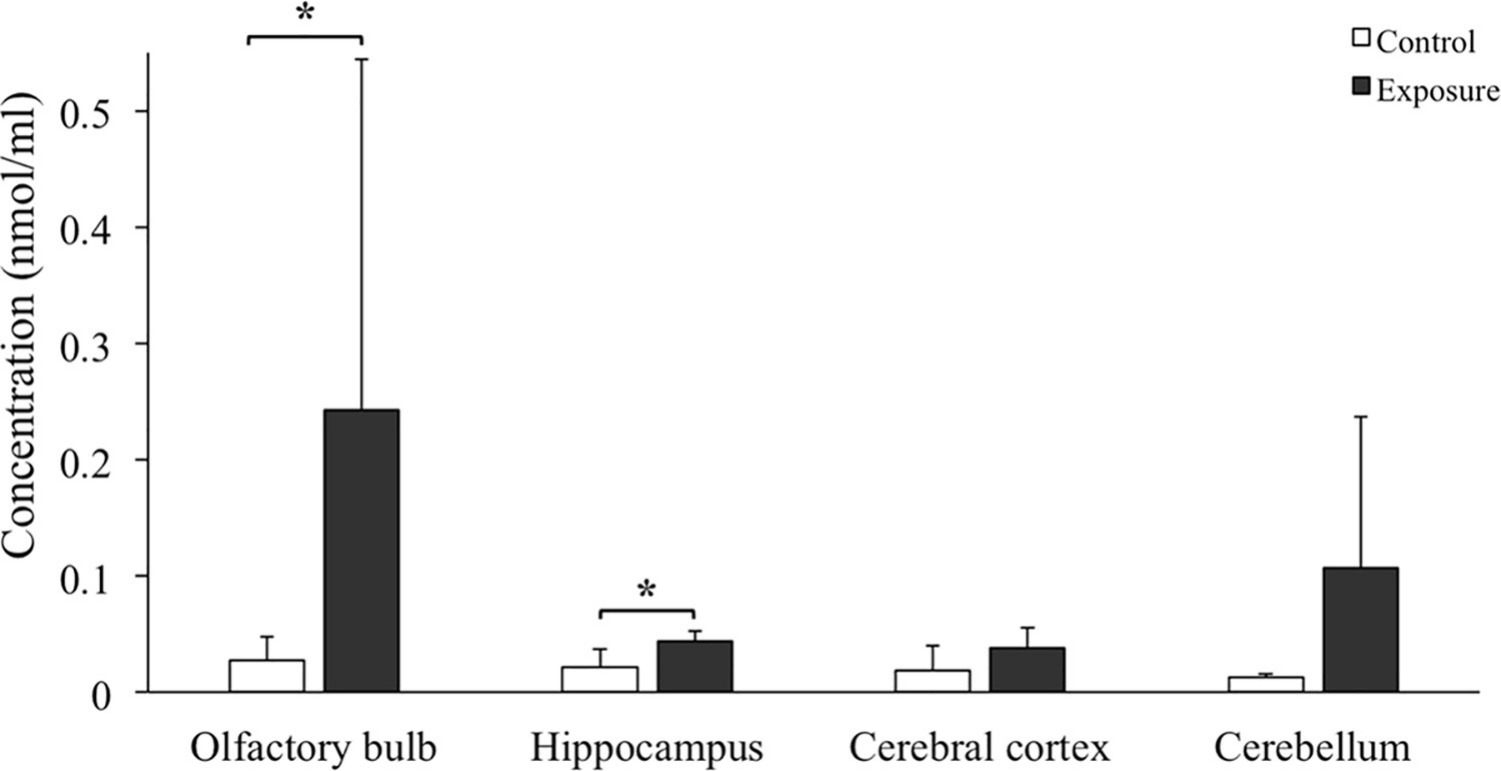
The MDA levels of individual AD mouse brain regions after PM_2.5_ exposure (n = 6) compared to control mice (n = 7). The values are presented as mean *±* SD. * indicates the significant differences between two groups with a *p* value lower than 0.05.

Fig 6 shows the effects of oxidative stress on the cortex, hippocampus, olfactory bulb, and cerebellum of mice exposed to PM_2.5_ (n = 6) or filtered air (n = 7). The levels of MDA in the hippocampus and olfactory bulb were significantly higher in the PM_2.5_-exposed group than the control group, and no obvious alterations were found in the cortex and cerebellum.

## Discussion

Compared to the PM_2.5_-induced pulmonary and cardiovascular adverse effects, PM_2.5_-mediated brain damage has become an emerging issue for human health. Previous epidemiological and animal studies revealed an association between PM_2.5_ exposure and AD [11, 12, 14]. Elevated levels of amyloid β and tau phosphorylation in brain and other AD-related molecular and cellular alterations, such as mitochondrial dysfunction, synaptic deficits, impaired neurite growth, neuronal cell death, glial cell activation, neuroinflammation, neurovascular dysfunction, and increased oxidative stress are observed [30]. However, most animals were exposed to higher levels of PM concentrations (range from 65.7~468.0 μg/m^3^) [17, 18, 31], which is several times human exposure in the real world. Our previous studies used a continuous, whole-body exposure system to study the relationship between the real-world exposure of PM and brain defects near a highway [28]. These results revealed that autophagy activation, neuron histological changes, and increased oxidative stress and neuroinflammation were observed in rats after chronic exposure of PM. Additional results also showed that even low concentration (range 8.6~16.3 μg/m^3^) exposure to PM caused brain impairment in wild-type mice (unpublished data) and spontaneously hypertensive rats [32, 33]. However, data of neuronal toxicity and AD-related disease progression after PM_2.5_ exposure in AD transgenic mouse models were limited. Therefore, we used the same exposure system to study PM_2.5_-induced brain alterations in the AD mouse brains.

The mean concentration of PM_2.5_ was 11.38 μg/m^3^ during the entire exposure period in the present study, which was near the WHO guidelines and similar to our previous studies [22, 32, 33]. The composition analysis of our collected PM_2.5_ showed that SO_4_^2-^, NH_4_^+^, and NO_3_^-^ were the enriched ions, which originate from urban traffic or coal burning [34]. These results were consistent with our previous studies [22, 35] and other traffic-related PM exposure studies [31, 36, 37]. The major metal components of PM_2.5_, including Na, K, Ca, Zn, Fe, Al, and Mg, were also similar with previous studies [17, 38] and considered traffic-related air pollutants [39–41].

In the current study, some 3xTg-AD mice were dead in either PM exposure group (n = 3) or control group (n = 1) around exposure periods. Although reasons for these unexpected deaths were not clear, some potential factors may play roles on this phenomenon. In this study, the ages of mice were from six-month-old to nine-month-old around the whole exposure experiments. Previous studies have shown that obvious extracellular deposits of Aβ occurred at the six-month-old of mice [42]. The impaired glia function was also found with the increasing age of mice (seven-month-old) [42]. These age-related physiological alterations may affect the health of 3xTg-AD mice. In addition, the gas pollutants from the outdoor environment were also introduced into the chambers along with the PM exposure. Our previous researches revealed that CO, NO_2_, SO_2_, and ozone were detected in both chambers [22]. These environmental pollutants may have effects on both PM exposure and control group mice.

To evaluate the cognition function and motor ability after a 3-month exposure to PM_2.5_, the present study used the MWM and rotarod tests. Although our results demonstrated that mice showed differences in spatial learning ability between days, there were no differences in most cognitive indicators between the PM_2.5_ exposed- and control mice. These results suggest that subchronic exposure to environmental levels of PM_2.5_ did not induce prominent cognitive or motor dysfunction. Previous studies revealed negative performances in MWM [43, 44], Barnes maze task, force swimming [45], and novel object recognition (NOR) test [46] in rodent models after subchronic or chronic exposure to higher concentrations of PM. However, no obvious behavioral differences in the MWM [32, 47], Y maze, force swimming [38], or rotarod test [28] were found in rodents under relatively lower concentration exposure to PM. Therefore, a low dose of PM_2.5_ may not cause severe clinical cognitive or motor decline.

Some microstructural changes, such as neuronal loss, were observed in the cortex of AD mouse brains after exposure. A decreasing number of neurons was postulated as a common pathological phenomenon of neurodegenerative diseases and may play essential roles in neural signal transmission and memory formation. A previous study also demonstrated that a loss of cell number was found in certain cortex and hippocampal regions after PM_2.5_ exposure or PM_2.5_ and formaldehyde coexposure (Liu et al. 2017 [47]). Notably, a recent study also showed similar pathological phenomena in the cortex of mouse offspring after parental exposure to PM_2.5_ during pregnancy [48]. According to the abovementioned pathological evidence, the neuronal morphological alterations observed in our study suggest that subchronic exposure to environmental levels of PM_2.5_ induced certain neuronal dysfunction.

Our study also revealed more neuronal morphological changes, including GVD and LF, in specific cortex regions-entorhinal cortex after PM_2.5_ exposure. The entorhinal cortex, which is located between the hippocampus and other cortex regions, is the most heavily damaged cortex region at an early stage of AD [49, 50]. Previous studies also demonstrated that the olfactory neurons that enter the entorhinal cortex was a route for PM entry into the CNS [51, 52]. Therefore, certain early AD-like neurodegenerative changes were induced by ordinary environmental concentrations of PM_2.5_. However, the evidence of PM-induced neuronal dysfunction in the entorhinal cortex is limited. Future work should pay more attention to this issue to understand how PM exposure mediates AD development and pathology.

To understand how the subchronic exposure to environmental levels of PM_2.5_ affected the neuronal dysfunction in the development of AD pathology, the protein expression of AD biomarkers, including Aβ_42_, t-tau, and p-tau, were assessed in different AD mouse brain regions. Our results revealed that p-tau expression in the olfactory bulb was significantly increased after PM_2.5_ exposure, but no obvious changes in Aβ_42_, or t-tau were observed. This overexpression was not seen in other brain regions.

The olfactory bulb is a direct route for the transfer of particles to the CNS [53, 54]. Olfactory dysfunction is also an early sign of AD and Parkinson’s disease. Previous studies demonstrated several neuropathological and molecular changes, including disturbed homeostasis in the expression of pathological Aβ and tau, in the olfactory bulb of AD patients and animals [55, 56]. The accumulation of pathological proteins and the related neuronal dysfunction in the olfactory bulb were associated with the occurrence of neuropathology in other brain areas, which may be used an early biomarker for AD and other neurodegenerative diseases [57]. Our previous studies also showed the elevated p-tau expression in the olfactory bulb of spontaneously hypertensive rats subchronic exposed to low-level PM_2.5_ [32].

For the most ambiguous finding, we observed an accumulation of p-tau, but not Aβ_42_, in the olfactory bulb of exposed mice, and there were no significant changes of Aβ_42_ or tau expression in other brain regions. This result suggests that the very early toxic changes of PM_2.5_ entry from nasal airway may not be attributed to the amyloid cascade. This conclusion conflicts with previous studies. These changes may be attributed to the lower exposure concentration and not the longer time period needed to induce the overexpression of Aβ_42_ and hyperphosphorylated tau. Previous studies revealed that chronic exposure to a higher level of nanosized PM and traffic-related air pollution particulate matter (300–468 μg/m3 for 10–15 weeks) induced Aβ_42_ deposition in AD transgenic mouse cortex (Cacciottolo et al. 2017 [18], Cacciottolo et al. 2020 [36]), but acute exposure to a lower concentration of PM_2.5_ (100 μg/m^3^ for seven days) did not produce a significant increase in Aβ_42_ in C57BL/6J mouse cortex (Liu et al. 2017 [47]). A study of wild-type rodents exposed chronically (six to nine months) to low levels of PM_2.5_ and PM_1_ also showed no obvious overexpression of tau and hyperphosphorylated tau in the cortex, hippocampus, or cerebellum (Bhatt et al. 2015 [17], Shih et al. 2018 [28]). Therefore, our results also support the conclusion of no obvious Alzheimer pathology after subchronic exposure to environmental levels of PM_2.5_. However, recent studies revealed that other modifications of tau, such as acetyl-tau were also associated with the development of AD. The increased level of acetyl-tau affected the synapse and autophagy function associated with the memory deficits in AD [58, 59]. Thus, the assessments of acetyl-tau expression after PM exposure in the future researches were needed.

To evaluate the possible mechanism of microglia activation and autophagy function in PM-related neurotoxicity, the present study assessed the expression of Iba1 and LC3B. Western blotting found decreased Iba-1 protein expression in the mouse cortex after PM_2.5_ exposure. However, no obvious changes of Iba-1 expression were observed in the IHC analysis. Although recent studies revealed that PM-related air pollutants induced the elevation of Iba-1 in the rodent species [60, 61], these studies were performed using much higher exposure concentrations than the current study. Because our exposed animals were middle-aged AD transgenic mice, the perturbations of microglia activation were likely due to the natural aging process in mice. These results suggest that subchronic exposure to environmental levels of PM_2.5_ may not induce obvious changes in microglia activation, which may be inferred by the aging process in the AD population. However, other markers of microglia activation, such as CD11b, were not examined. Therefore, more assessments of microglia activation are needed to evaluate the subchronic effects of environmental levels of PM_2.5_ exposure in future studies. However, the expression of LC3B was not different between the PM_2.5_ exposure and control AD mouse brains. Similarly, the observed no obvious differences in brain LC3 protein expression between PM exposure mice and control mice in the current study were similar to a previous study that assessed subchronic exposure (three months) of environmental levels of PM_1_ in a mouse model [28]. Therefore, alterations in microglia activation and autophagy function may not be prominently induced under lower exposure concentrations and shorter exposure duration to PM_2.5_, as shown in our study.

There was a significant increase of MDA levels in the AD mouse brains, especially in the olfactory bulb and hippocampus after a 3-month exposure to PM_2.5_ in our study. MDA is an end product of the lipid peroxidation chain reaction, and it is used as an indicator to evaluate the oxidative stress level in brain disease. Brains are composed of various lipids, including polyunsaturated fatty acids, which are rich in double bonds that are easily attacked by ROS. The accumulation of MDA in the brain suggests increasing lipid peroxidation and disturbances in brain lipid hemostasis. Previous studies demonstrated that the increase in oxidative stress was a prominent early event that mediated AD pathogenesis [62, 63]. Therefore, our current observations revealed that subchronic exposure of environmental levels of PM_2.5_ induced oxidative stress in AD mouse brains. Similar results were found in rodent brains after exposure to PM, diesel exhaust, and dusty PM [43, 60, 64].

Our results also demonstrated that the PM_2.5_-induced oxidative stress was more obvious in the olfactory bulb and hippocampus than the cortex and cerebellum. A previous study also revealed that acute exposure of diesel exhaust (250–300 μg/m^3^ for six hours) induced an increase in MDA levels in various mouse brain regions, especially the olfactory bulb and hippocampus [60]. Another study demonstrated that markers of oxidative stress, such as 4-hydroxy-2-nonenal (4-HNE) and 3-nitrotyrosine (3-NT), increased rapidly in the olfactory bulb and olfactory neuroepithelium of mice after subchronic exposure to nanoscale PM (343 μg/m^3^ for 45 cumulative hours over three weeks) but no obvious oxidative alterations in the cortex or cerebellum [31]. These variances in brain region sensitivity may be partially explained because that PM, or its absorbed components, reach the hippocampus and striatum directly via the olfactory bulb then spread around the cortex via transneuronal transport [53, 65]. Accompanied with the PM transportation to different brain regions, PM induced oxidative stress and neuroinflammation. Therefore, the early exposure route of PM, including the olfactory bulb and hippocampus, may show higher oxidative stress than other brain regions. Low concentration exposure to PM may not trigger obvious oxidative stress. Chronic exposure to environmental levels of PM_2.5_ (yearly average 11.70 μg/m^3^ for nine months) in a mouse model also did not show obvious oxidative stress in the cortex as assessed the level of HNE-adduct [17]. Therefore, subchronic exposure to environmental levels of PM_2.5_ (average 11.38 μg/m^3^ for three months) in the present study may only induce oxidative stress in the mouse olfactory bulb and hippocampus and not in the cortex or cerebellum.

**Fig 7:**
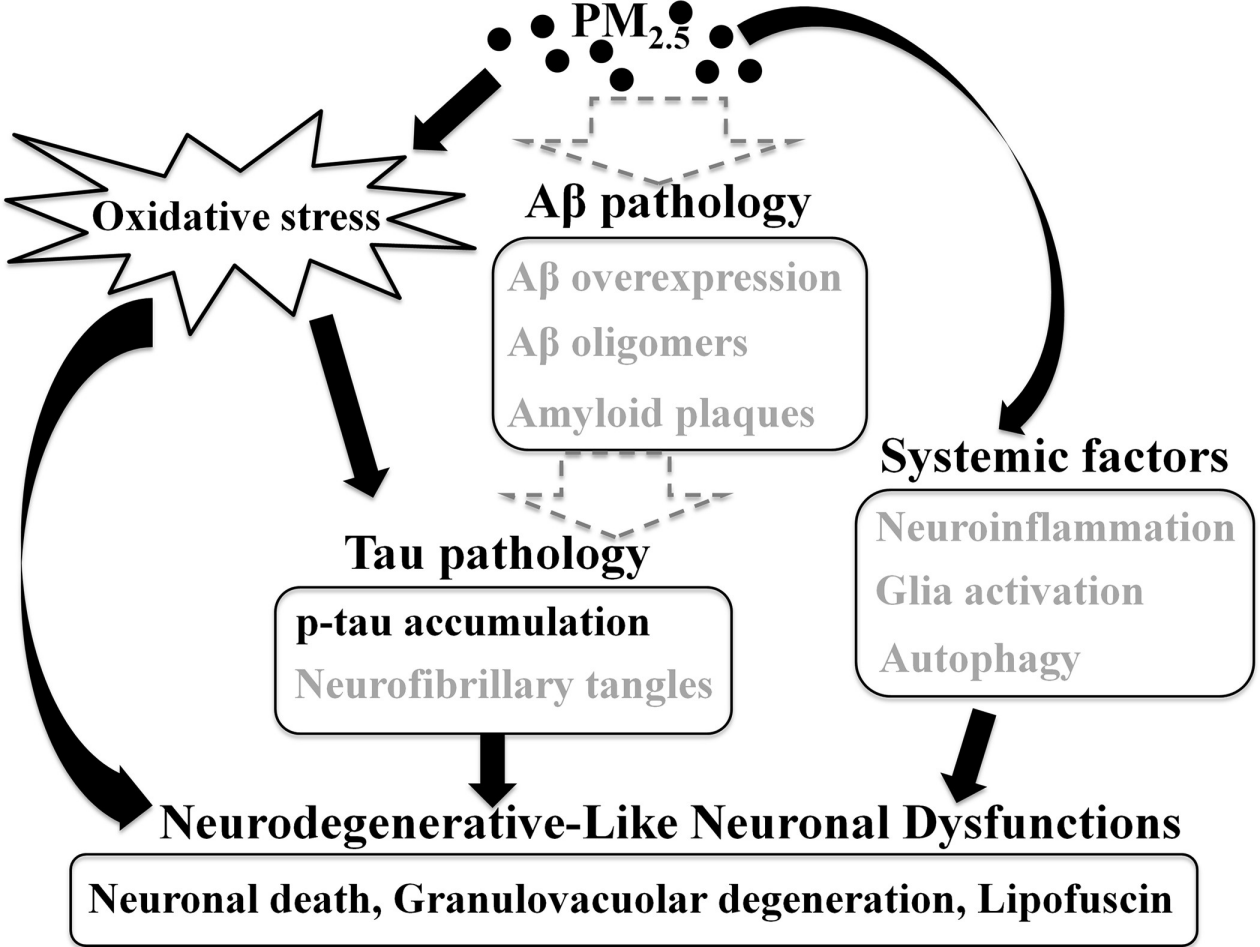
The possible mechanisms of subchronic exposure to environmental levels of PM_2.5_-mediated neuronal dysfunction in AD mouse brains. The black words and arrows indicated the observations (such as neuronal death and p-tau accumulation) and their biological roles found in this study. The gray parts were described the PM-mediated evidences and mechanisms reported from previous researches, but not in the current studies.

In summary, our results showed that subchronic exposure to PM_2.5_ induced a possible pathway of neurodegenerative-like neuronal dysfunction (Fig 7). Exposure to environmental levels of PM_2.5_ induced enough oxidative stress to drive further neuronal exacerbation. This oxidative stress may have directly or indirectly (via tau pathology) induced the neuronal loss and abnormal morphology. However, activation of the Aβ-dominated neuronal pathological cascade was not observed in the current exposure condition. This result may be attributed to our shorter exposure period and lower exposure concentration because higher exposure concentration or longer exposure time of PM_2.5_ triggered the amyloid cascade in previous studies [18, 36]. Neuron loss was also found in the whole cortex. This result means that the neuronal dysfunction may not be induced only by the olfactory bulb-hippocampus-cortex route. The systemic route, via the pulmonary-cardiac circulation, may contribute the neuron damage in the other cortex areas. Therefore, these dose-dependent and two different mediated pathways played important roles in the brain toxicity of mice under a lower exposure concentration and shorter period of PM_2.5_ exposure (Fig 7).

To the best of our knowledge, this report is the first study to examine the neuronal dysfunction induced by environmental levels of PM_2.5_ in AD transgenic mice. Most PM-induced animal studies were performed in wild-type rodent species. ApoE knockout mice (Kleinman et al. 2008 [66]) and J20-hAPPswe mice (Cacciottolo et al. 2020 [36]) were used to assess AD-related toxicity (Table 3). Poly-transgenic mouse models, including 3xTg-AD [66], E3 and E4FAD [18], were used recently to study PM-mediated AD development and pathology. These studies demonstrated that PM exposure induced detriments of spatial learning, reference and short-term memory, and increased the accumulation of Aβ and tau proteins, related molecular and cellular changes and neuronal damage. These results showed that models were powerful tools to study PM-induced central neuronal system toxicity.

**Table 3:** PM-induced neuronal dysfunction on brains of AD disease models.

Year	Particle type and source	Mouse	Sex	Age	Exposure condition	Conc.	Observation	Citation
				Start ~ end		(μg/m^3^)		
2008	CAPs collected near freeway I-10 in the Los Angeles, CA	APOE^-/-^	M	1.5-month-old ~	5 h/day	30.4	Increased AP1, NF-κB, GFAP, and pJNK/JNK ratio in cortical tissue after either lower or higher exposure.	[67]
				3-month-old	3 days/week	114.2		
					6 weeks			
2017	nPM collected near highway 101 in the Los Angeles, CA	E3FAD	F	2-month-old ~	5 h/day	468.0	1. Increased Aβ protein load and oligomers in the cortex of E4FAD.	[18]
		E4FAD		7-month-old	3 days/week			
							2. Selective neuronal atrophy (lower neurite density) in the CA1 of hippocampus of E3FAD and wild type mice.	
					15 weeks			
							3. Decreased synaptic proteins (GluR1) in the hippocampus of all three types of mice.	
2019	UFP collected near roadway in Rochester, NY	3xTg-AD	M	12.5-month-old ~	4 h/day	57.0	1. Protracted detriments in spatial learning, reference memory, and short-term memory independent of underlying AD genotype.	[66]
				13.0-month-old	4 days/week			
					2 weeks			
							2. UFP worsened olfactory discrimination in 3xTgAD mice	
2020	nPM collected near highway I-110 in the Los Angeles, CA	J20-hAPPswe	M	Unknown	5 h/day	300.0	1. Increased Aβ_40_, Aβ_42_, and Aβ load n the cortex as well as increased APP in the lipid raft of the cortex.	[36]
					3 days/week			
					10 weeks			
							2. Increased lipid oxidation (4-HNE) in the cortex.	

CAPs: concentrated ambient particles; GFAP: glial fibrillary acidic protein; UFP: ultrafine particulate matter; nPM: nano-scale PM; E3FAD: 5xFAD^+/ −^ /human APOE ε3/ε3; E4FAD: 5xFAD^+/ −^ /human APOE ε4/ε4; GluR1: glutamate receptor subunit

Some potential issues existed in previous studies. First, all previous studies used a noncontinuous PM exposure system. Although this type of exposure system controls the exposure condition better, the impact underlying this treatment may not well reflect the real exposure conditions of the human population. Second, most observations from previous AD models were restricted to higher concentrations (30~ 468 μg/m^3^) of PM exposure, which were three-fold higher than the WHO guidelines. The current study used continuous exposure of real-world outdoor air that was near the WHO guideline for PM exposure values during our exposure periods. The neuronal dysfunction and toxicity observed under this exposure condition may more accurately correlate with the real exposure situation. The exposure age and gender were different between the present and previous studies. Previous AD mouse studies were exposed to PM at an earlier age (< 2-month-old) [18, 67] or aged mouse (12.5-month-old) [66]. Increased activator protein 1 (AP1), nuclear factor κB (NF-κB), glial fibrillary acidic protein (GFAP), and β-amyloid and decreased neurite density and synaptic proteins (GluR1) were found in young mouse brains after PM exposure, and the older mice showed behavior dysfunction and worsened olfactory discrimination after PM exposure. However, the effects of PM exposure at the adult stage are not clear. Our results suggested that PM exposure in mature adult and middle ages 3xTg-AD mice induced several neurodegenerative-like neuronal dysfunctions. Most previous studies [36, 66, 67], except one study [18] (Table 3), used male AD mice to study PM-induced AD toxicity. One previous study demonstrated that knowledge of gender-specific effects of PM on AD toxicity were required because a higher prevalence existed in women than men [66]. Therefore, the results of the present study provide more evidence about PM-induced effects in less discussed populations.

Although certain PM-induced neuronal dysfunctions were observed in the current study, there were some limitations should be addressed. In this study, we examined the subchronic effects of PM exposure in the middle-aged 3xTg-AD mice. However, previous studies showed that the multiple memory deficits and olfactory discrimination were observed in the aged (12.5 months) male 3xTg-AD and non-transgenic mice after a short-term inhalation exposure (two weeks) to ultrafine PM [66]. These results highlighted the potential adverse effects happened in the older mice in response to PM exposure. Therefore, more studies for PM-mediated neuronal toxicity in the older mice are necessary. On the other hand, the PM-mediated effects were assessed only in the female population in the current study. More studies are needed to study the subchronic effects of low-level of PM_2.5_ exposure in male mice. Next, the assessments of Aβ expression could be improved. In this study, we studied the Aβ pathology by the histopathology and western blot technologies. In addition to western blot, the ELISA tests were also widely used to examine Aβ expression in AD-related studies [17, 18, 36]. Thus, combined the western bolt and ELISA methods to determine the Aβ performance in the future works may provide more complete picture to understand PM-mediated effects in the AD development. Finally, certain within-group variability was existed in the results of western blot and MDA analysis. This phenomenon may be attributed to the following reasons. In this study, the main exposure is the environmental level of PM_2.5_. Since the exposure is near the WHO guidelines, the effects of PM_2.5_ may not be obvious like previous researches which conducted much higher doses of PM_2.5_ [18, 36]_._ Under this low-level exposure, the physiological differences of the individual mouse may interfere our observations. In addition, limited sample size in this study (n = 6~7) may also contribute this variability. When the sample size was limited, the outlier value from certain individual increased the variability within group. More animals for each group may alleviate this problem.

In conclusion, our current results showed that subchronic exposure to environmental levels of PM_2.5_ caused neuronal dysfunction, particularly increased oxidative stress and p-tau, neuronal cell loss, granulovacuolar degeneration, lipofuscin, and neuroinflammation, in the certain brain regions of 3xTg-AD mice. Our study did not observe the amyloid-related changes shown in previous studies, which suggests that the AD-like toxicity induced by PM_2.5_ exists under the condition of higher density of acute exposure or chronic accumulation. This result means a gradience-dependent toxicity of PM_2.5_, as shown in Fig 7. These neurodegenerative-like changes were primarily observed in the cortex, especially the entorhinal cortex region, hippocampus and olfactory bulb, which indicated the important entry route of environmental PM_2.5_, the spreading pathway of oxidative damage, and the other impact route from the systemic circulation. More prolonged effects of PM_2.5_-mediated neurodegenerative disease, including AD, are needed in future studies.

## Supporting information

S1 File The supplementary figures (S1-S7 Figs in S1 File) and tables (S1 and S2 Tables in S1 File). (PDF)

S1 Raw images (PDF)
