# Supplementary material for: Three month inhalation exposure to low-level PM2.5 induced brain toxicity in an Alzheimer’s disease mouse model
Source: PLoS One. 2021 Aug 26;16(8):e0254587. doi: 10.1371/journal.pone.0254587 (PMC8389369; doi:10.1371/journal.pone.0254587)
Supplement: S1 File — (PDF) [file pone.0254587.s001.pdf]

S1 File. The supplementary figures (S1-S7), tables (S1-S2), and raw western blot images.

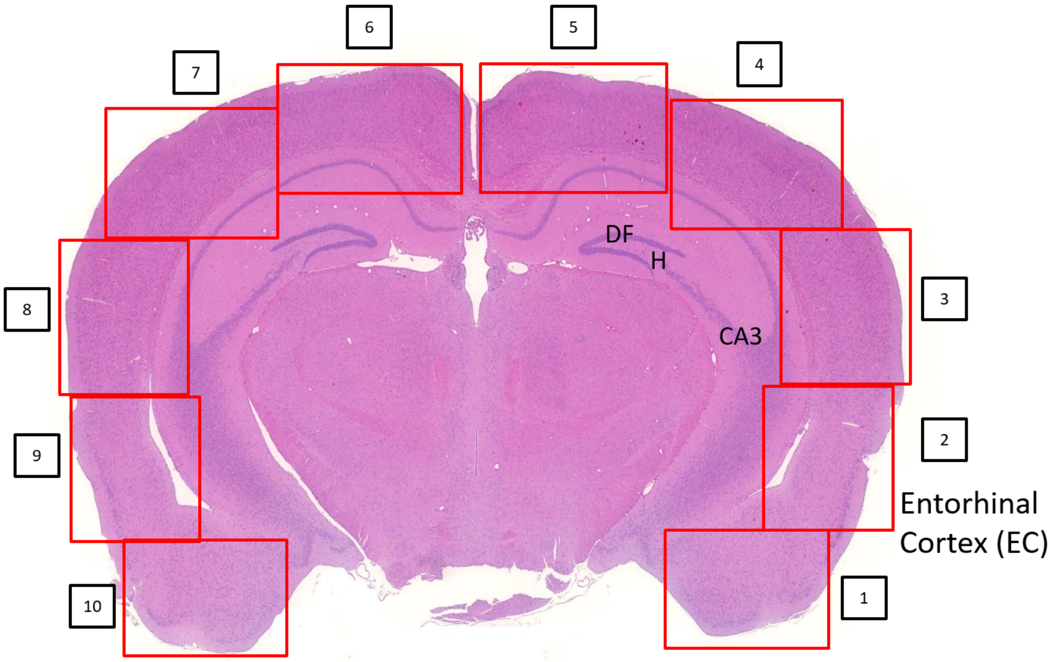

S1 Fig. The representative view of mouse cortex for neural cell counting.

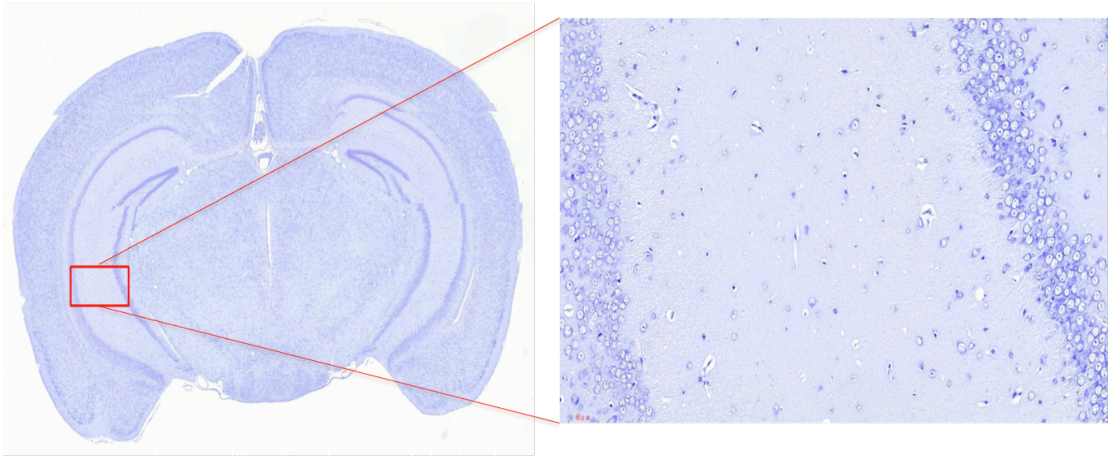

S2 Fig. The representative view of mouse hippocampus for neural cell counting.

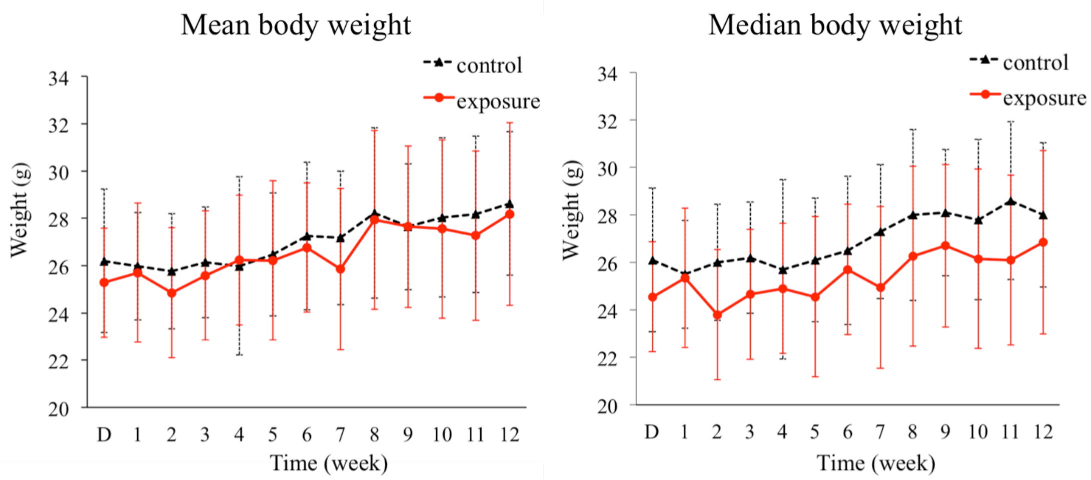

S3 Fig. The boy weight of AD mice around three-month exposure period.

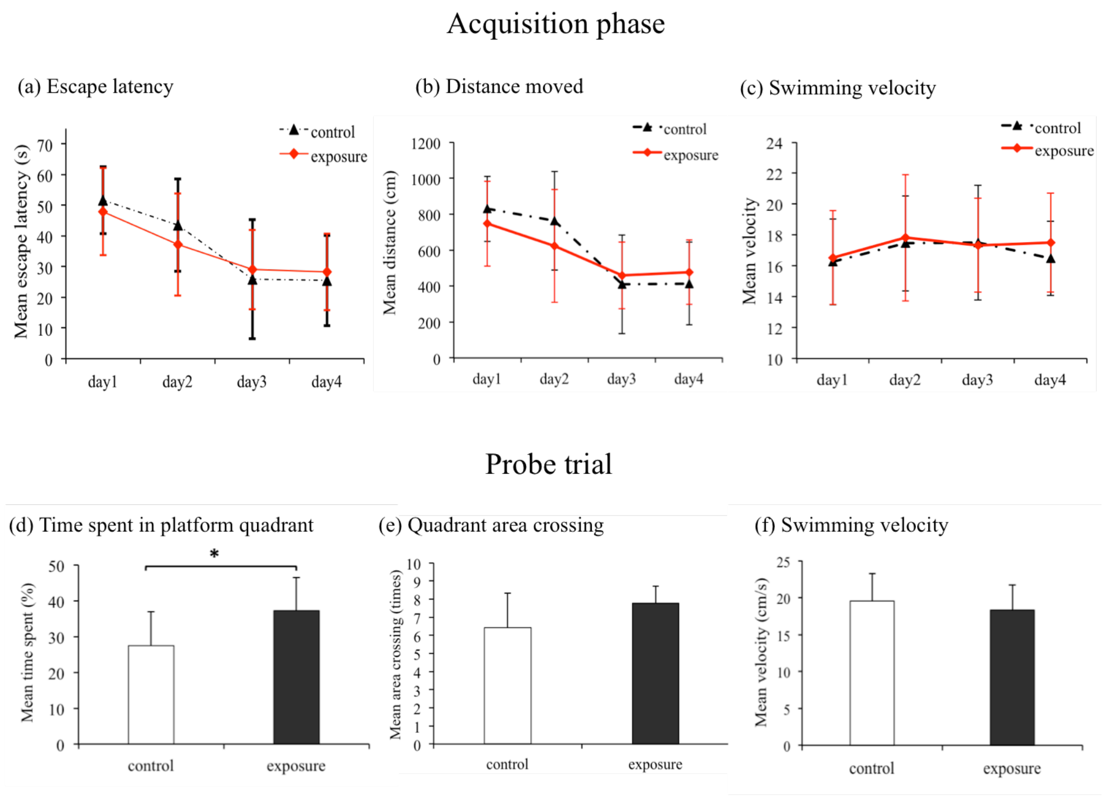

S4 Fig. **The behavior performance of Morris Water Maze test after exposure.** The average escape latency (a), distance moved (b), and swimming velocity (c) for the acquisition phase (the first 4 days) as well as the average time spent in platform quadrant (d), quadrant area crossing (e), and swimming velocity (f) for the probe trial (the 5th day).

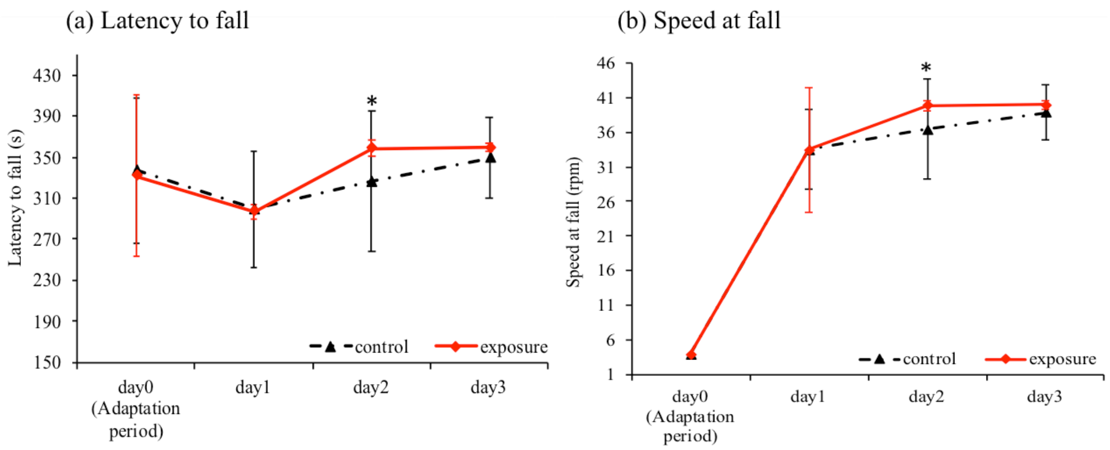

S5 Fig. The behavior performance of rotarod test after exposure. The average latency to fall (a) and speed at fall (b) around four days.

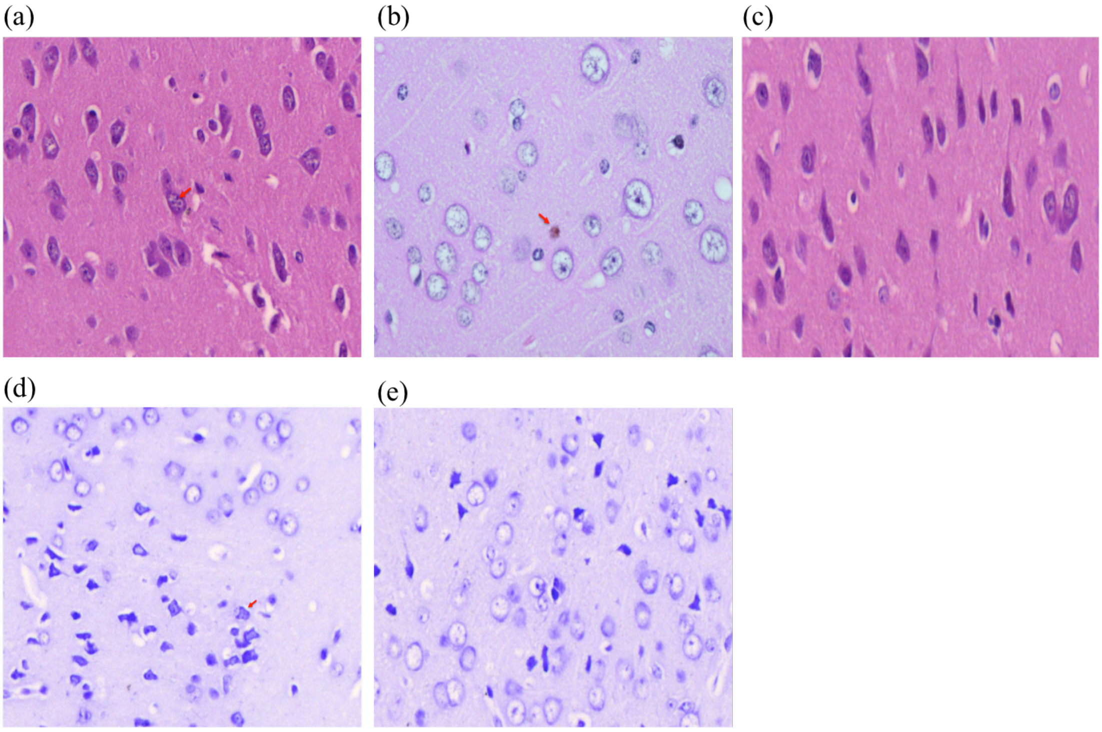

S6 Fig. The histopathological views of neuronal morphological alterations in the AD mouse brains (400X). In the representative H&E images, the granulovacuolar degeneration (a) and lipofuscin (b) were found in the $PM_{2.5}$ exposure group, but not in the control group (c). The results from nissl staining also showed the granulovacuolar degeneration in the certain $PM_{2.5}$-exposed mouse brain (d), but not in the control mouse brain (e).

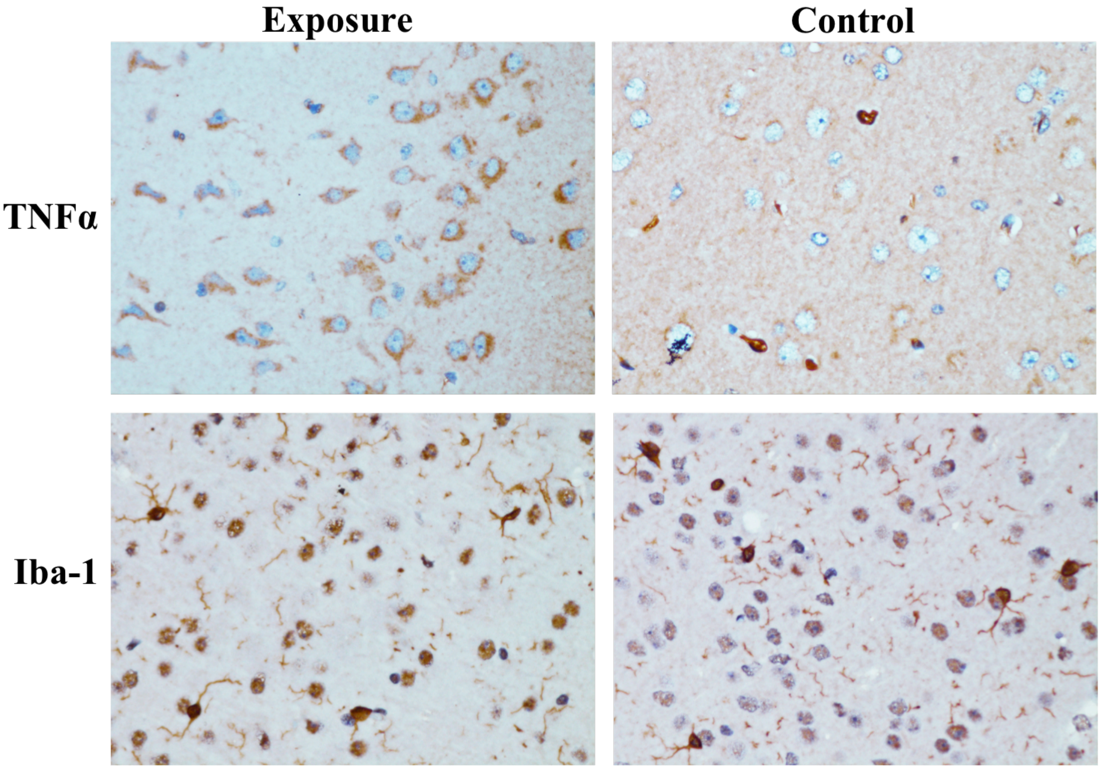

S7 Fig. The histopathological views of IHC staining (400X) of TNFα and Iba-1 in the cortex of AD mice in the $PM_{2.5}$ exposure or control group.

S1 Table. The total metal ion composition of the $PM_{2.5}$ around exposure period.

	Mean (ng/m^3)	Median (ng/m^3)	SD (ng/m^3)	Min (ng/m^3)	Max (ng/m^3)
Al	66.4	72.6	27.4	28.7	110.7
Fe	94.5	91.3	30.3	64.5	152.5
Na	165.8	168.5	32.6	119.4	229.6
Mg	38.9	37.7	12.2	22.8	59.6
K	159.5	154.8	101.9	53.2	368.3
Ca	282.9	254.0	101.8	170.9	511.4
Sr	1.3	0.8	2.2	0.4	6.9
Ba	3.0	2.1	4.4	1.0	14.5
Ti	5.8	5.8	2.0	4.0	10.4
Mn	8.6	8.3	2.9	4.6	13.2
Co	0.1	0.1	0.0	0.1	0.1
Ni	4.0	3.6	1.7	2.4	7.3
Cu	5.7	4.3	2.9	4.2	12.6
Zn	20.2	18.0	5.9	13.3	31.7
Mo	0.5	0.4	0.2	0.3	1.0
Cd	0.2	0.2	0.1	0.1	0.5
Sn	2.1	2.0	0.9	1.0	3.4
Sb	0.9	0.7	0.5	0.4	2.2
Tl	0.1	0.0	0.0	0.0	0.1
Pb	10.2	7.1	5.7	3.6	21.3
V	5.9	5.7	1.7	3.6	8.7
Cr	3.0	2.7	1.2	2.2	5.8
As	0.9	0.8	0.3	0.4	1.5

S2 Table. **The water-soluble metal ion composition of the $PM_{2.5}$ around exposure period.**

	Mean (ng/m^3)	Median (ng/m^3)	SD (ng/m^3)	Min (ng/m^3)	Max (ng/m^3)
Al	10.5	10.5	7.0	4.1	25.3
Fe	17.3	20.0	8.3	6.8	27.7
Na	107.8	116.0	15.6	77.8	130.8
Mg	16.5	16.6	7.0	9.4	33.7
K	124.3	138.8	72.5	45.7	278.9
Ca	31.3	23.1	19.3	17.7	74.3
Sr	0.9	0.4	2.1	0.2	6.4
Ba	2.3	1.2	3.9	0.8	12.5
Ti	0.5	0.4	0.2	0.3	0.8
Mn	4.6	4.8	1.9	2.3	7.6
Co	0.0	0.0	0.0	0.0	0.0
Ni	1.2	1.2	0.4	0.7	2.0
Cu	3.1	2.6	2.3	1.5	8.6
Zn	23.7	22.9	7.1	15.4	37.5
Mo	0.2	0.2	0.1	0.1	0.4
Cd	0.2	0.1	0.1	0.1	0.4
Sn	0.3	0.3	0.2	0.1	0.6
Sb	0.5	0.4	0.3	0.1	1.0
Tl	0.0	0.0	0.0	0.0	0.1
Pb	5.7	4.0	3.6	1.5	13.4
V	3.7	3.6	1.3	1.7	5.8
Cr	0.4	0.4	0.1	0.2	0.7
As	0.7	0.7	0.3	0.3	1.1
