# Supplementary material for: Three month inhalation exposure to low-level PM2.5 induced brain toxicity in an Alzheimer’s disease mouse model
Source: PLoS One. 2021 Aug 26;16(8):e0254587. doi: 10.1371/journal.pone.0254587 (PMC8389369; doi:10.1371/journal.pone.0254587)
Supplement: S1 Raw images — (PDF) [file pone.0254587.s002.pdf]

ECL images in the **Olfactory bulb**

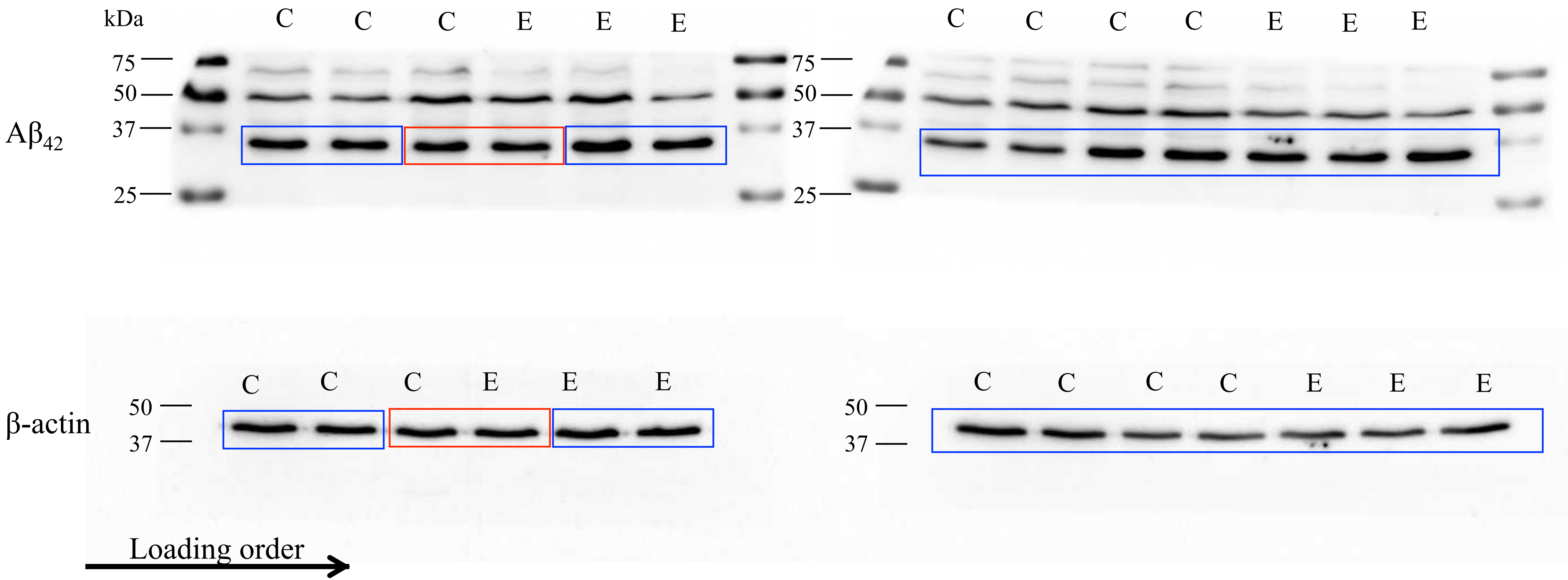

C: control; E: exposure

The **selected area** were used in the statistics and **shown** in the manuscript as the representative images

The **selected area** were used in the statistics, but the images were **not shown** in the manuscript

ECL images in the **Hippocampus**

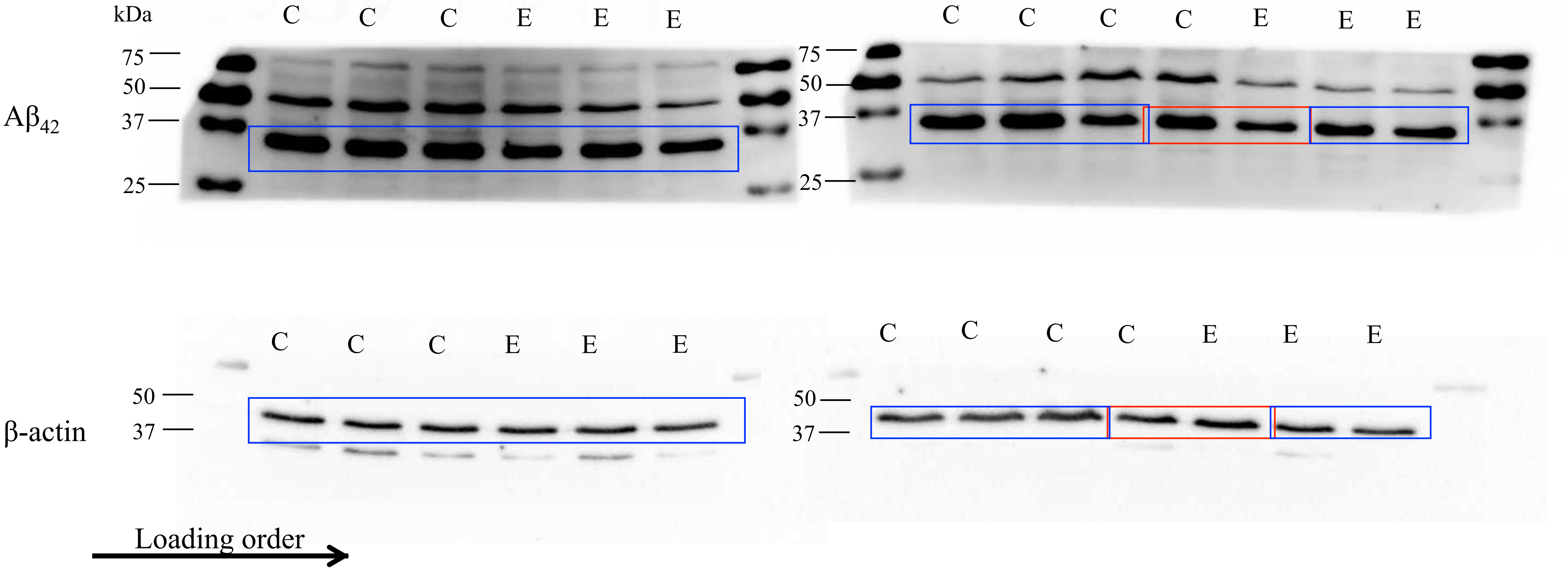

C: control; E: exposure

The **selected area** were used in the statistics and **shown** in the manuscript as the representative images

The **selected area** were used in the statistics, but the images were **not shown** in the manuscript

ECL images in the **Cerebral cortex**

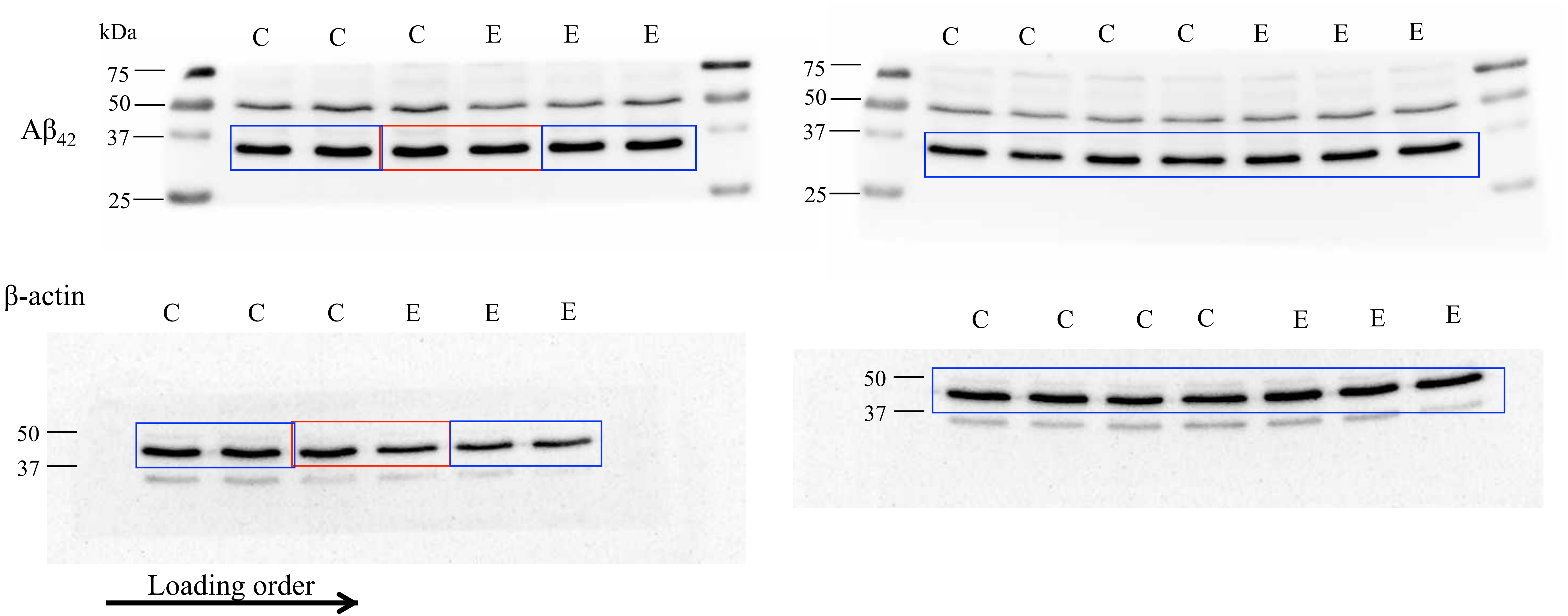

C: control; E: exposure

The **selected area** were used in the statistics and **shown** in the manuscript as the representative images

The **selected area** were used in the statistics, but the images were **not shown** in the manuscript

ECL images in the **Cerebellum**

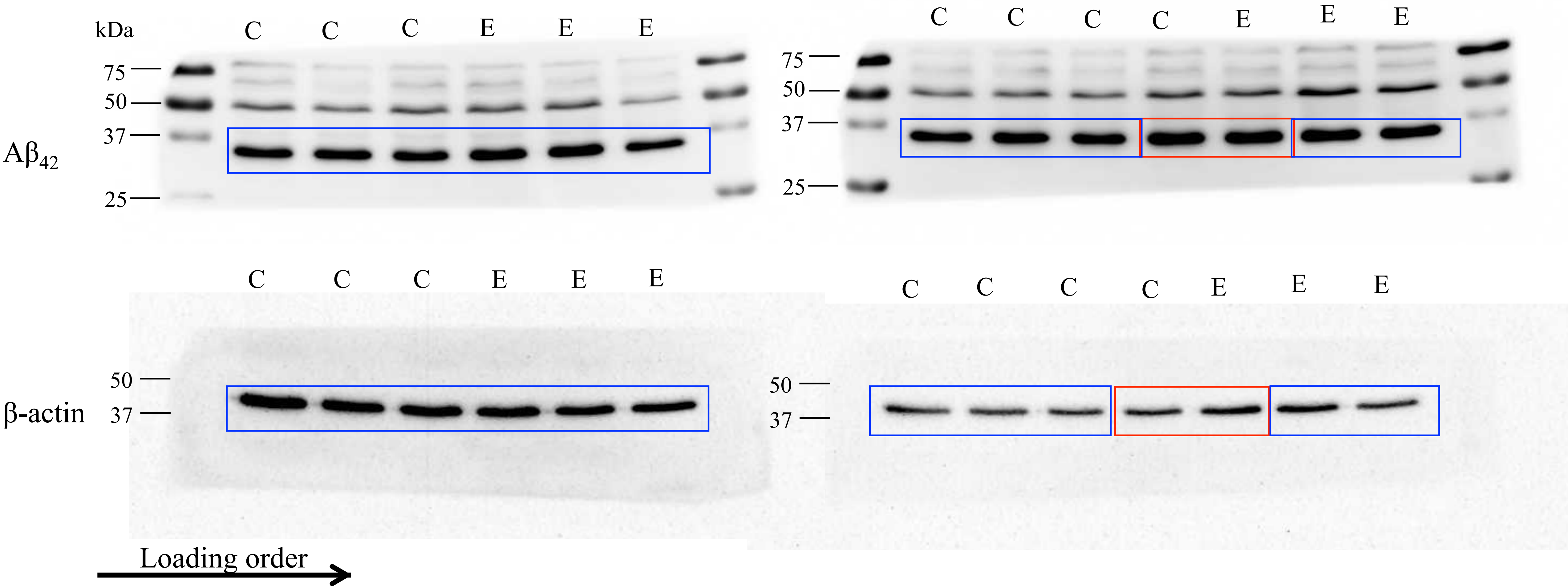

C: control; E: exposure

The **selected area** were used in the statistics and **shown** in the manuscript as the representative images

The **selected area** were used in the statistics, but the images were **not shown** in the manuscript

ECL images in the **Olfactory bulb**

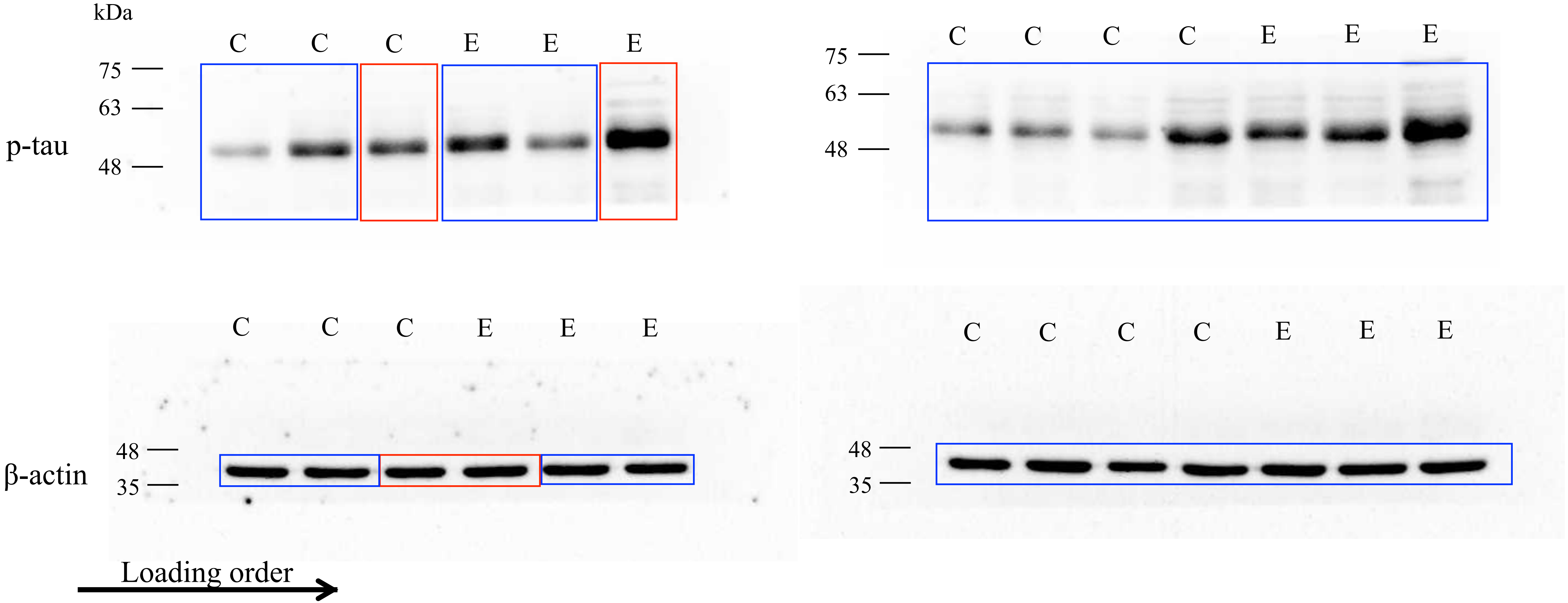

C: control; E: exposure

The **selected area** were used in the statistics and **shown** in the manuscript as the representative images

The **selected area** were used in the statistics, but the images were **not shown** in the manuscript

ECL images in the **Hippocampus**

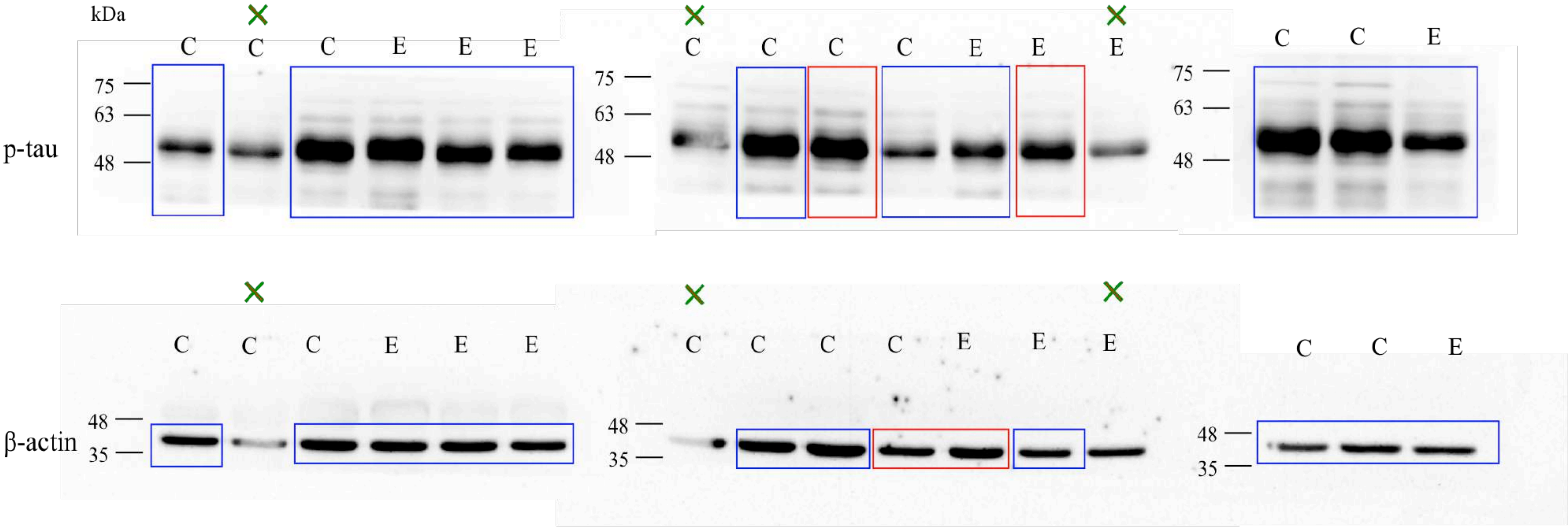

Loading order →

C: control; E: exposure

The **selected area** were used in the statistics and **shown** in the manuscript as the representative images

The **selected area** were used in the statistics, but the images were **not shown** in the manuscript

× were **not used** in the statistics and **not shown** in the manuscript

ECL images in the **Cerebral cortex**

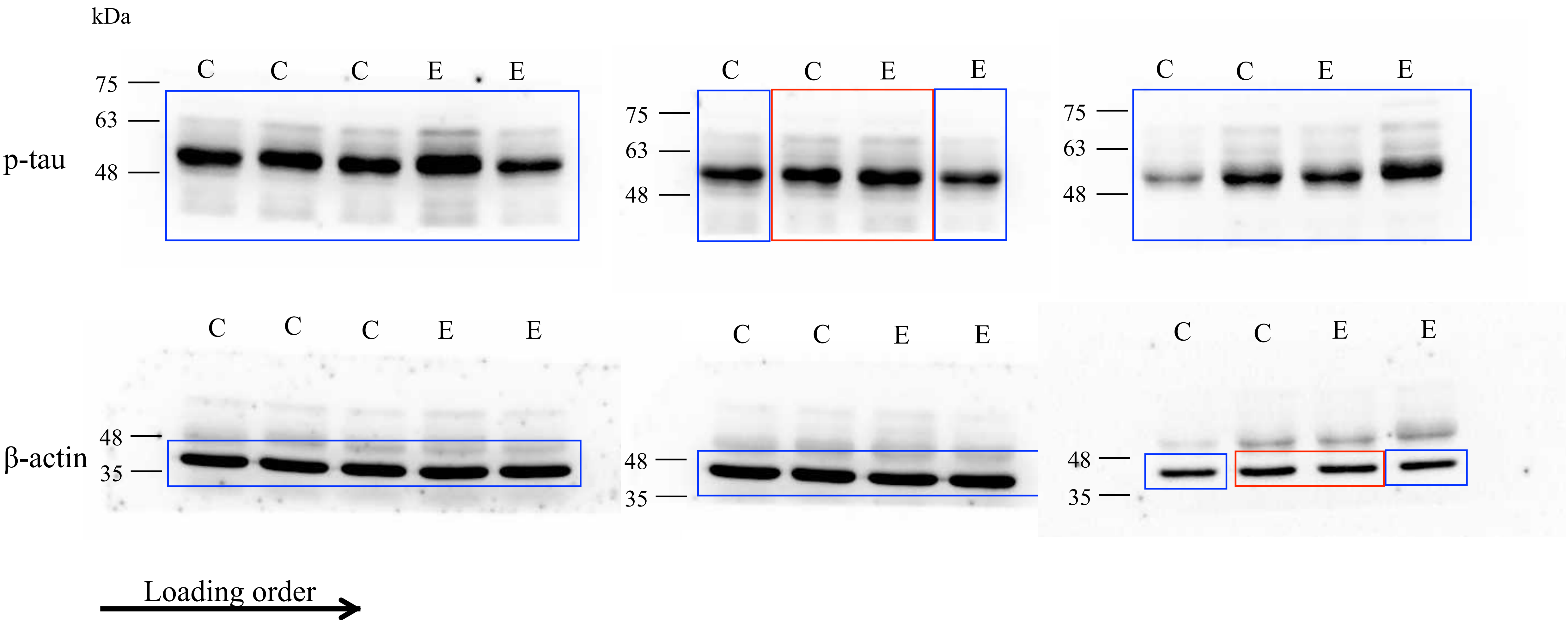

C: control; E: exposure

The **selected area** were used in the statistics and **shown** in the manuscript as the representative images

The **selected area** were used in the statistics, but the images were **not shown** in the manuscript

ECL images in the **Cerebellum**

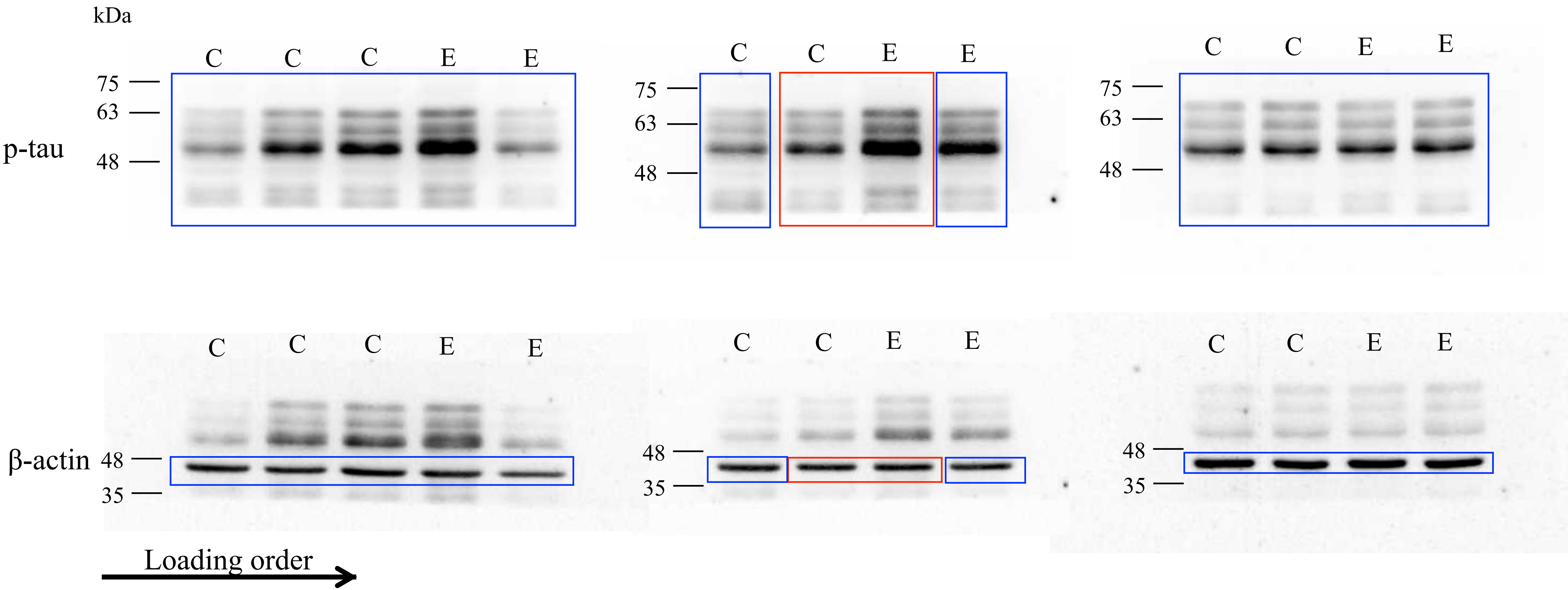

C: control; E: exposure

The **selected area** were used in the statistics and **shown** in the manuscript as the representative images

The **selected area** were used in the statistics, but the images were **not shown** in the manuscript

ECL images in the **Olfactory bulb**

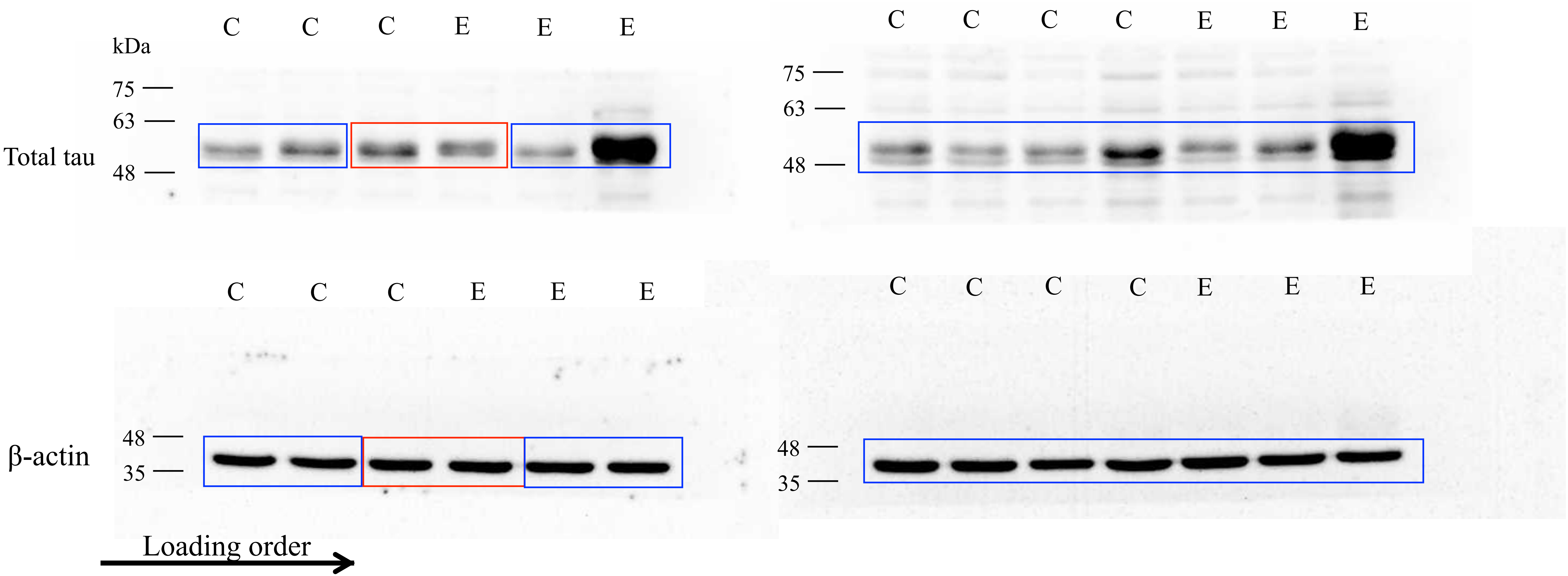

C: control; E: exposure

The **selected area** were used in the statistics and **shown** in the manuscript as the representative images

The **selected area** were used in the statistics, but the images were **not shown** in the manuscript

ECL images in the **Hippocampus**

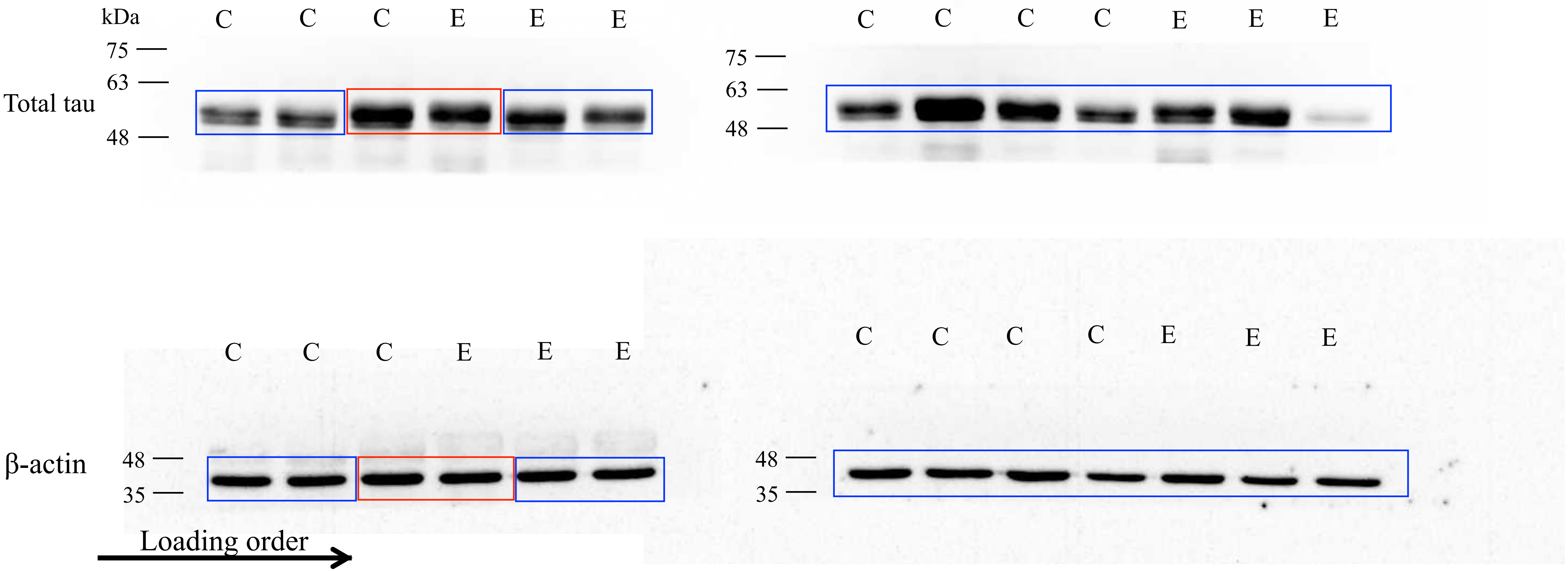

C: control; E: exposure

The **selected area** were used in the statistics and **shown** in the manuscript as the representative images

The **selected area** were used in the statistics, but the images were **not shown** in the manuscript

ECL images in the **Cerebral cortex**

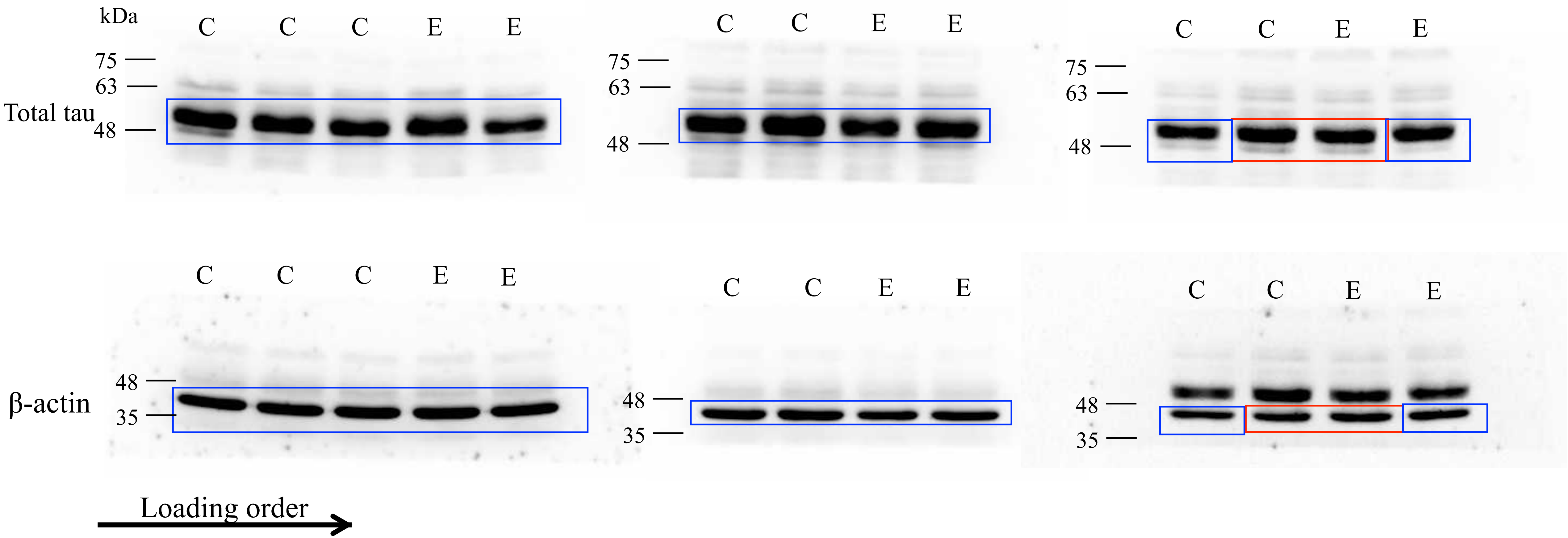

C: control; E: exposure

The **selected area** were used in the statistics and **shown** in the manuscript as the representative images

The **selected area** were used in the statistics, but the images were **not shown** in the manuscript

ECL images in the **Cerebellum**

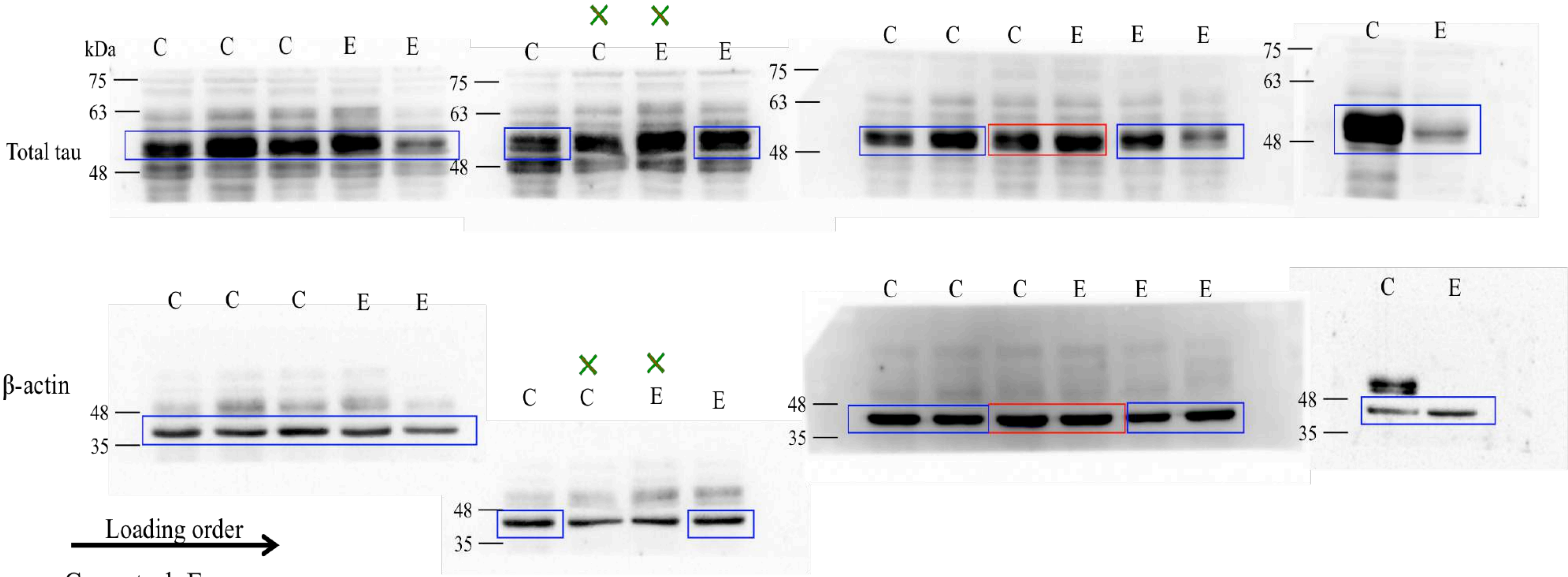

C: control; E: exposure

The **selected area** were used in the statistics and **shown** in the manuscript as the representative images

The **selected area** were used in the statistics, but the images were **not shown** in the manuscript

× were **not used** in the statistics and **not shown** in the manuscript

ECL images in the **Olfactory bulb**

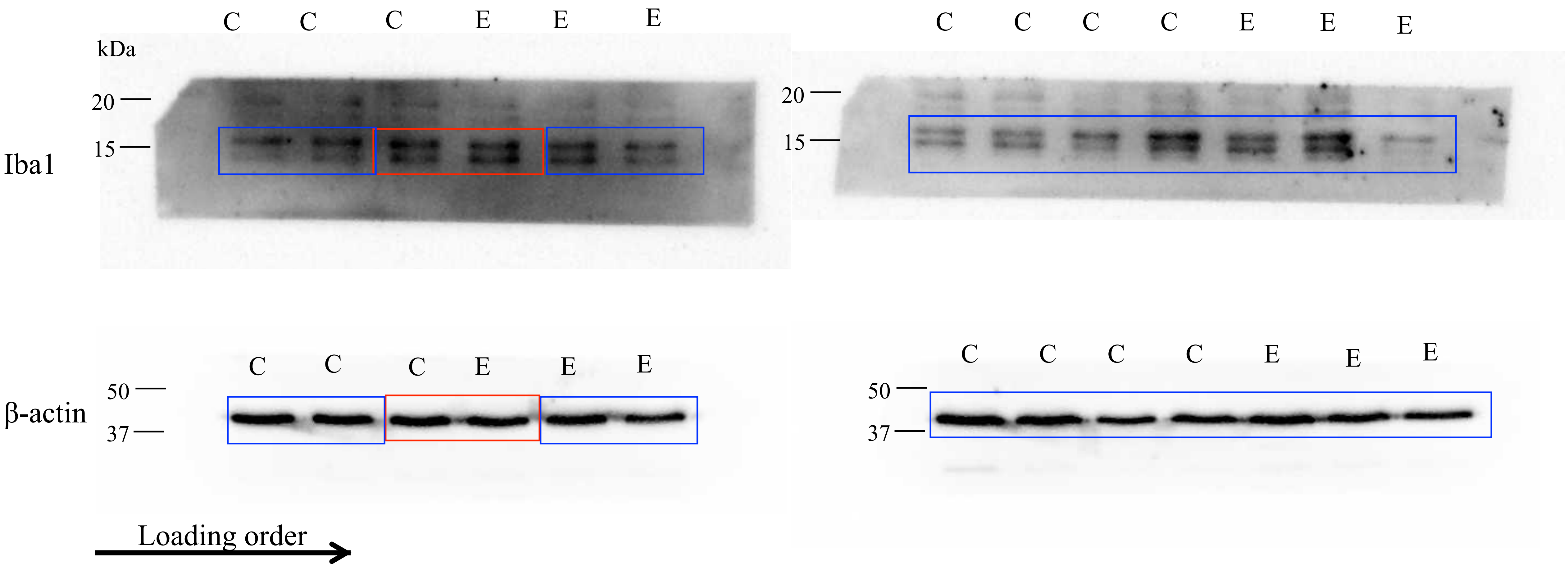

C: control; E: exposure

The **selected area** were used in the statistics and **shown** in the manuscript as the representative images

The **selected area** were used in the statistics, but the images were **not shown** in the manuscript

ECL images in the **Hippocampus**

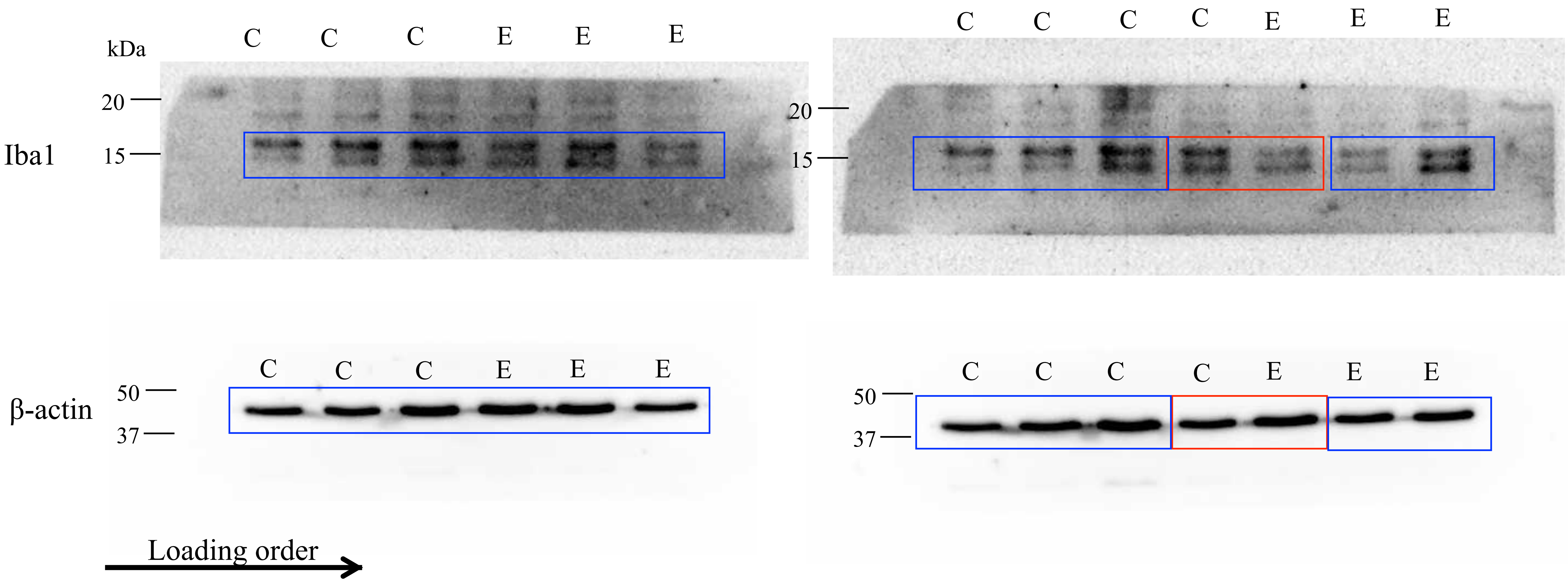

C: control; E: exposure

The **selected area** were used in the statistics and **shown** in the manuscript as the representative images

The **selected area** were used in the statistics, but the images were **not shown** in the manuscript

ECL images in the **Cerebral cortex**

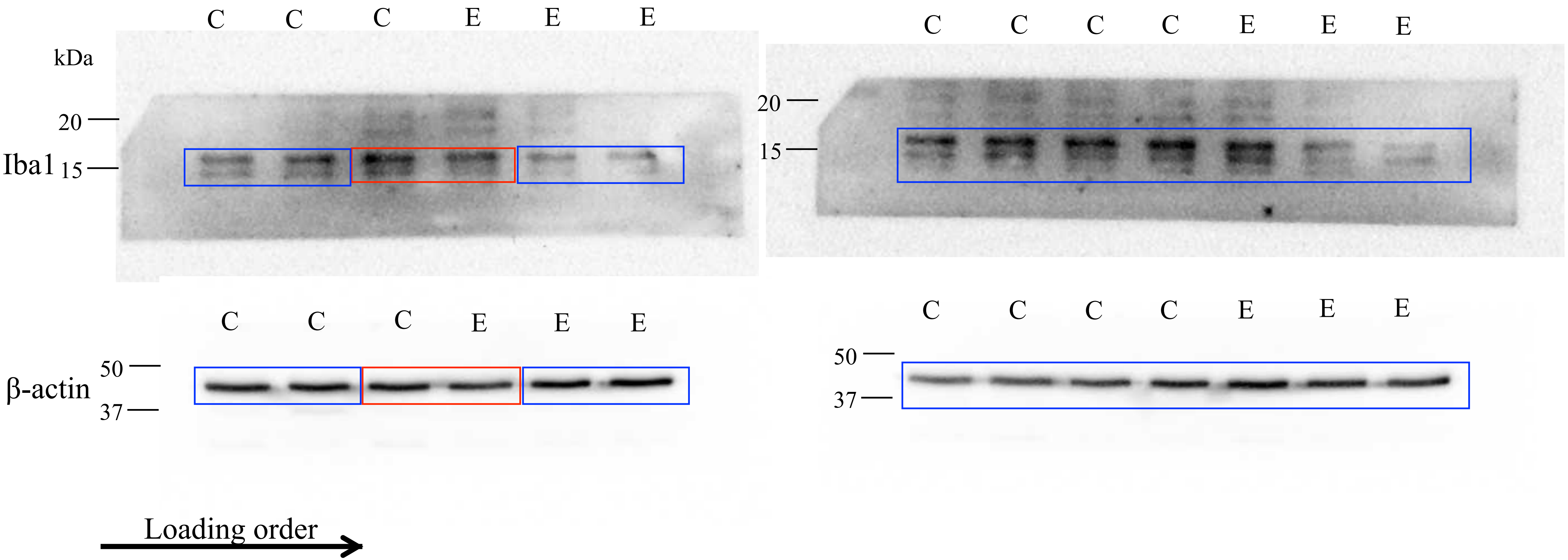

C: control; E: exposure

The **selected area** were used in the statistics and **shown** in the manuscript as the representative images

The **selected area** were used in the statistics, but the images were **not shown** in the manuscript

ECL images in the **Cerebellum**

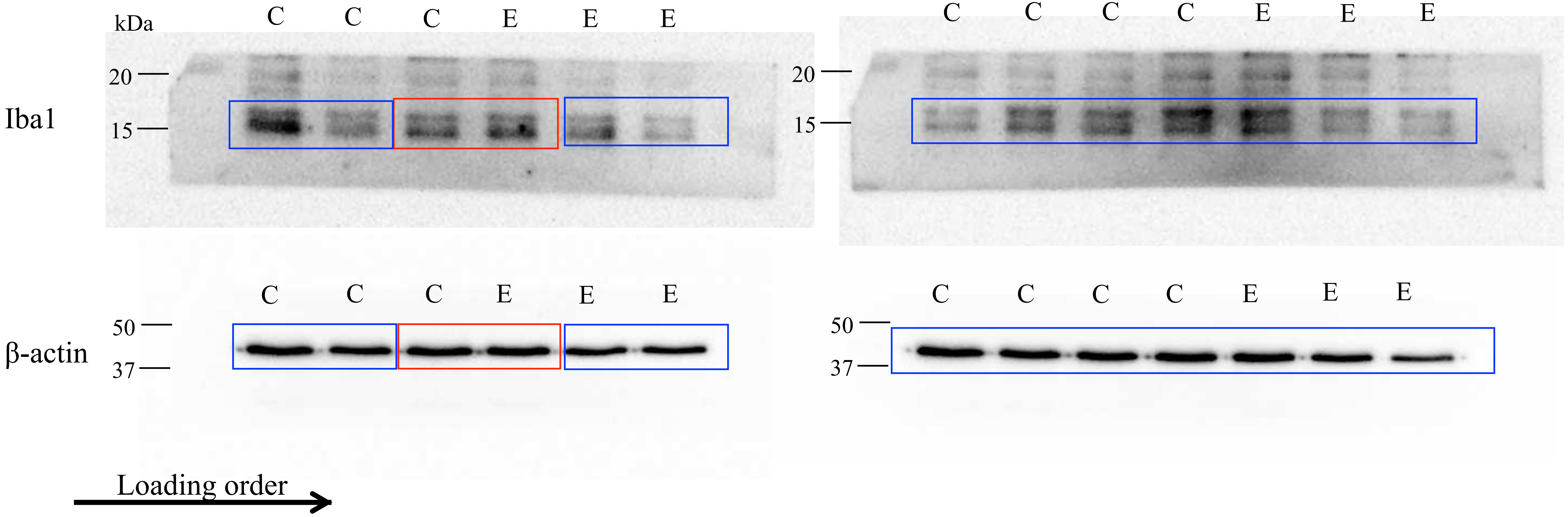

C: control; E: exposure

The **selected area** were used in the statistics and **shown** in the manuscript as the representative images

The **selected area** were used in the statistics, but the images were **not shown** in the manuscript

ECL images in the **Olfactory bulb**

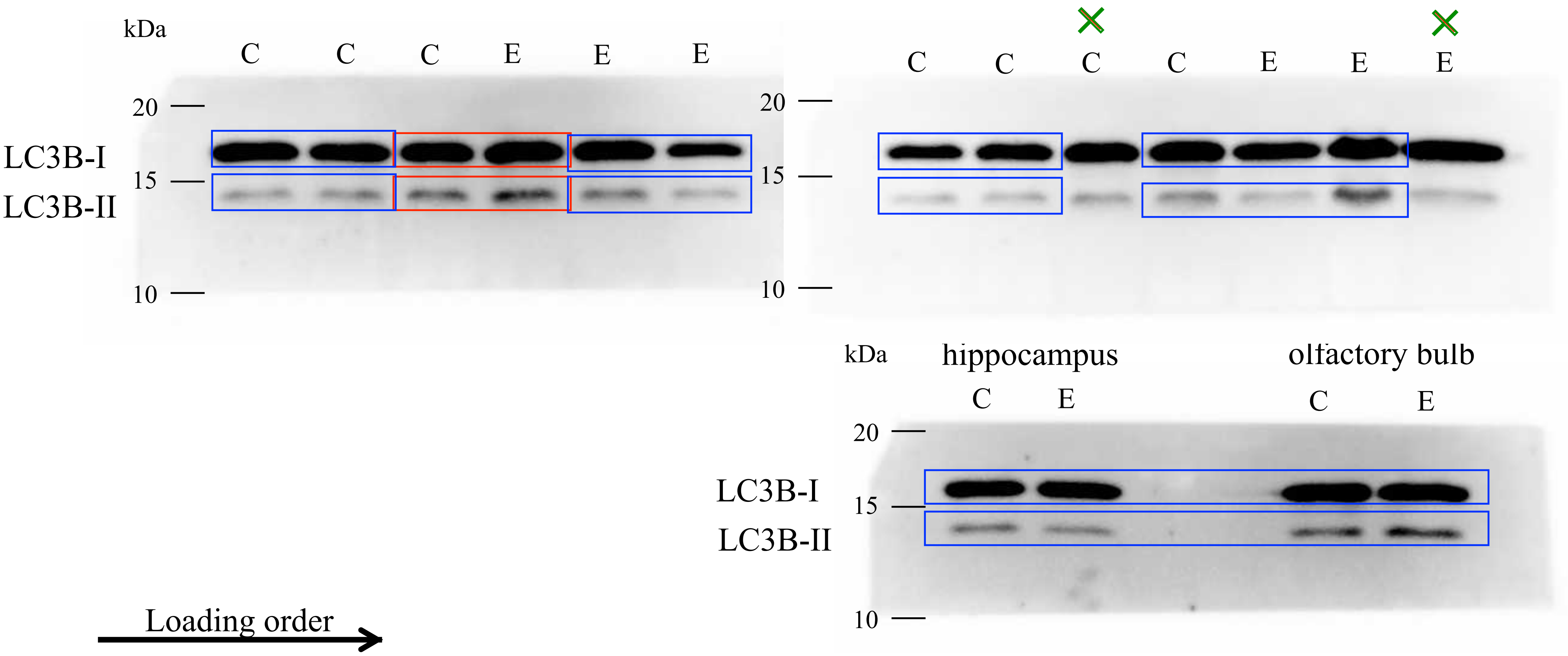

C: control; E: exposure

The **selected area** were used in the statistics and **shown** in the manuscript as the representative images

The **selected area** were used in the statistics, but the images were **not shown** in the manuscript

✕ were **not used** in the statistics and **not shown** in the manuscript

ECL images in the **Hippocampus**

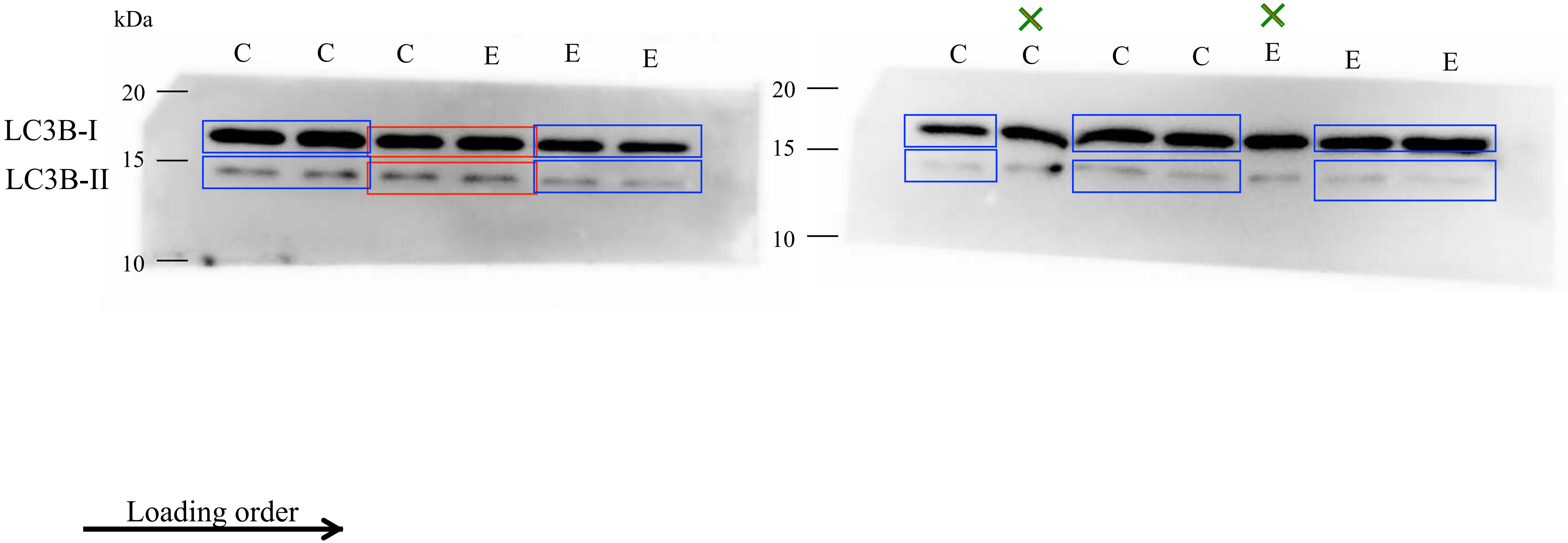

C: control; E: exposure

The **selected area** were used in the statistics and **shown** in the manuscript as the representative images

The **selected area** were used in the statistics, but the images were **not shown** in the manuscript

× were **not used** in the statistics and **not shown** in the manuscript

ECL images in the **Cerebral cortex**

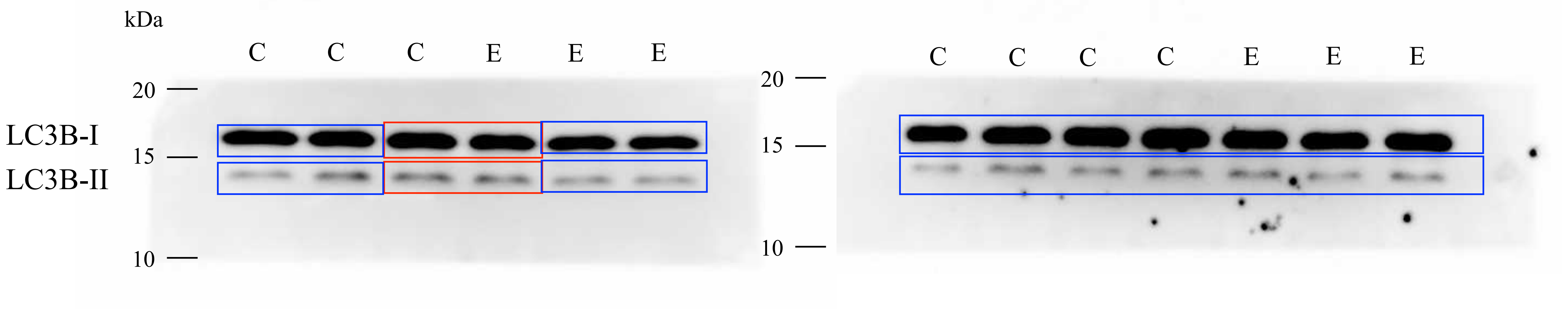

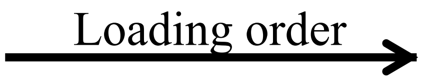

C: control; E: exposure

The **selected area** were used in the statistics and **shown** in the manuscript as the representative images

The **selected area** were used in the statistics, but the images were **not shown** in the manuscript

ECL images in the **Cerebellum**

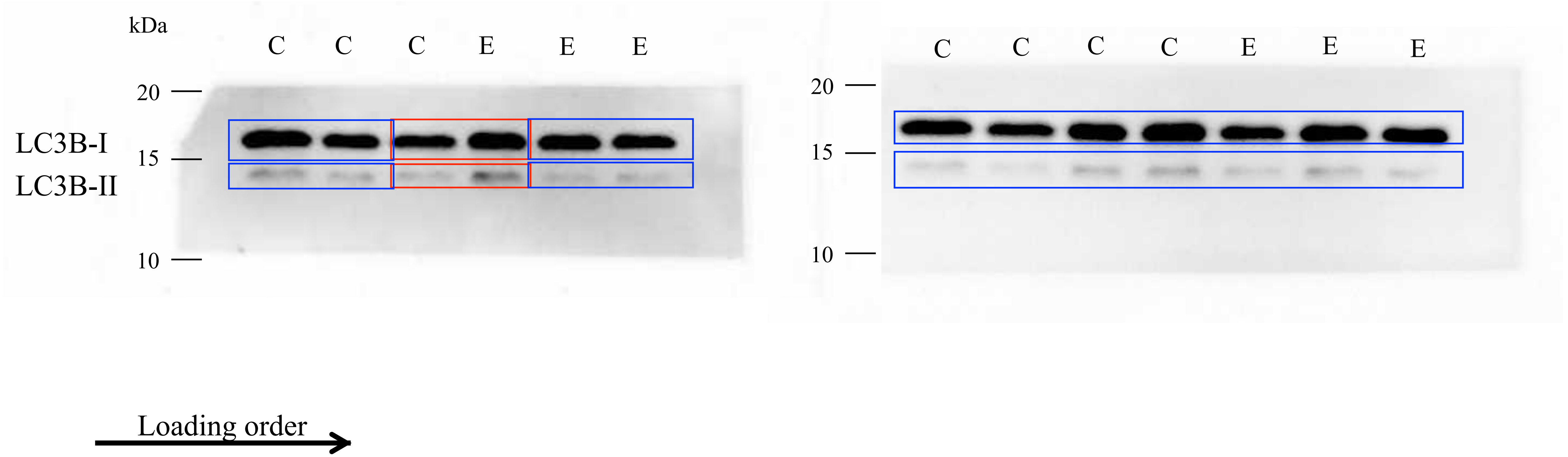

C: control; E: exposure

The **selected area** were used in the statistics and **shown** in the manuscript as the representative images

The **selected area** were used in the statistics, but the images were **not shown** in the manuscript
